# Catalpol: an natural multifunctional iridoid glycoside with promising therapeutic properties

**DOI:** 10.3389/fmolb.2026.1768334

**Published:** 2026-02-18

**Authors:** Guannan He, Jing Song, Ruixuan Ma, Yixing Zhou, Yamin Xue, Farong Zhang

**Affiliations:** 1 College of First Clinical Medical, Shandong University of Traditional Chinese Medicine, Jinan, China; 2 Shandong Yuze Pharmaceutical Industry Technology Research Institute Co., Ltd., Dezhou, China; 3 Department of Nephrology, Affiliated Hospital of Shandong University of Traditional Chinese Medicine, Jinan, China

**Keywords:** catalpol, new dosage form, novel drug delivery routes, pharmacological activity, safety

## Abstract

Catalpol, an iridoid glycoside predominantly derived from the fresh or dried root tuber of *Rehmannia glutinosa* Libosch (a member of the *Scrophulariaceae* family), it is a representative compound with the highest content in *Rehmannia glutinosa* Libosch, and it is also a key index component for evaluating the quality of *Rehmannia glutinosa* Libosch. Since 2005, it has been continuously included in various editions of China Pharmacopoeia. In this review, we collected relevant data from the Web of Science, PubMed, China National Intellectual Property Administration and China Knowledge Resource Integrated databases in recent 5 years. Catalpol exhibits a broad range of therapeutic effects, addressing various diseases through intricate mechanisms. These include organ- and tissue-protective actions on the kidneys, bones, nervous system, heart, brain, liver, lungs, uterus, ovaries, and more, alongside notable anti-arthritis, anti-cancer, and anti-diabetic properties. The protective mechanisms of catalpol primarily involve its anti-inflammatory, antioxidative stress, anti- or pro-apoptotic, anti-fibrotic, metabolism-regulatory, anti-endoplasmic reticulum stress (ERS), and pyroptosis-modulating functions. Furthermore, catalpol influences a variety of signaling pathways, cells, and molecules, and through these multifaceted actions, it achieves its maximal therapeutic potential. In recent years, the development of different targeted drug delivery formulations and administration routes of catalpol maximise its efficacy has become a major focus of research. What’s more worth mentioning is that “catalpol tablets”, a new class I Chinese medicine developed on the basis of this monomer component, has been approved to enter the clinical trial stage in China. However, in-depth investigation is required to elucidate the mechanisms of action of catalpol, and more clinical trials are required to assess the clinical value of this compound.

## 1 Introduction

**FIGURE 1:**
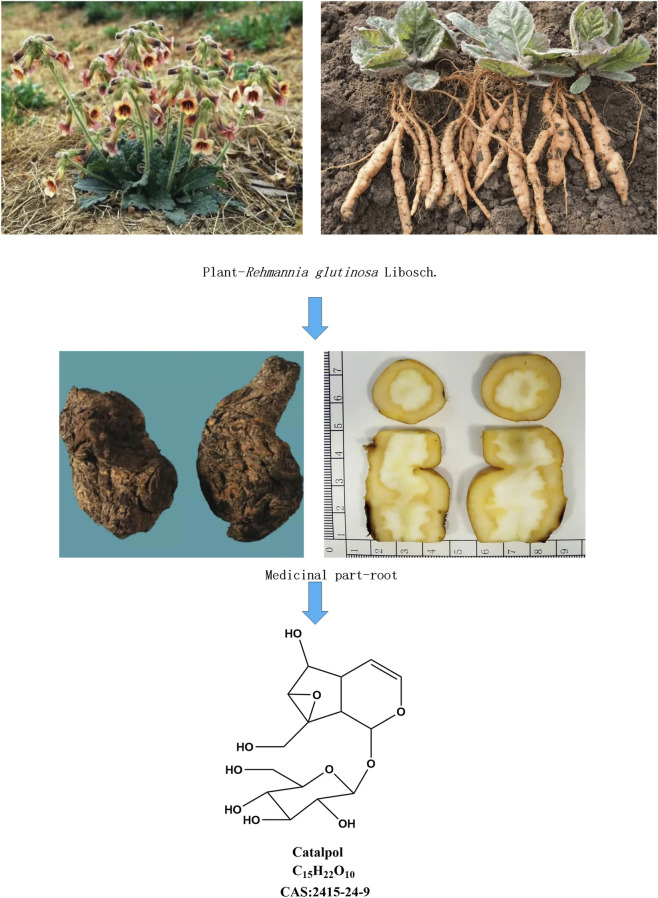
*Rehmannia glutinosa* Libosc*h* plants, *Rehmannia glutinosa* Libosch medicinal material, chemical structures of catalpol.

*Rehmannia glutinosa* is the fresh or dried root tuber of *Rehmannia glutinosa* Libosch, a member of the *Scrophulariaceae* family. It was first recorded in Shennong’s Classic of Materia Medica. Recorded in China Pharmacopoeia, it is often used to treat consumptive thirst, namely, thirst and dry mouth caused by diabetes. At the same time, it also has a good improvement effect on kidney inflammation and fibrosis. It is a safe and commonly used traditional Chinese medicine. At present, it is also listed in the homology catalogue of medicine and food in China. Catalpol, an iridoid glycoside extracted from *Rehmannia glutinosa*, is characterized by its polar structure, high solubility in water, and a molecular formula of C_15_H_22_O_10_ with a molecular weight of 362.45 (Lai, 2018). The content of catalpol serves as a quality control standard for *Rehmannia glutinosa* in the Chinese Pharmacopoeia (Figure 1).

In recent years, more and more natural compounds of Chinese herbal medicine have gradually become an important source of new drug research, development and an effective means of adjuvant therapy by virtue of their clear pharmacological activities, multi-target effects, safety advantages and the support of modern technology. Monomer of traditional Chinese medicine and its effective components have been paid attention to because of their clear chemical structure and exact curative effect, and play a great role in the settlement of many diseases. Studies have confirmed that organs and tissues are susceptible to damage from various internal and external factors in clinical, such as drugs and trauma, which lead to structural and functional impairments (Mattson, 2019; Licata, 2016; Zhang et al., 2017). The primary mechanisms of organs and tissues damage include inflammation, oxidative stress, cell apoptosis or pyroptosis, metabolic disturbances, and fibrosis. Catalpol, a naturally derived compound, has emerged as a potent agent with significant protective effects on damaged tissues and organs (Feng et al., 2019; Wei et al., 2019; Zou et al., 2019). Modern pharmacological research has confirmed catalpol’s broad spectrum of effects, including antioxidation, anti-inflammation, and anti-fibrosis, along with notable therapeutic benefits for conditions such as diabetes, nephropathy, and neurological disorders (Zhu and Wang, 2019; Jiao et al., 2020; Liu et al., 2018; Chen et al., 2019; Sun et al., 2019; Zhu et al., 2019). Due to its established clinical efficacy and the ongoing discovery of new pharmacological activities, catalpol has garnered widespread attention globally and has become a focus of active research in the medical field. Therefore, a comprehensive review of it not only helps to provide scientific theoretical basis for traditional Chinese medicine, but also provides a treasure house of resources for modern drug research and development, so as to provide more valuable reference for clinical application.

TCM is increasingly recognized for its low toxicity and minimal side effects. Mice with type 2 diabetes exhibit no toxic symptoms when treated with catalpol, and reasonable doses of catalpol have not shown significant adverse effects in rodents or humans (Zhang, 2024). *In vitro* studies have demonstrated that when L02 cells were cultured with varying concentrations of catalpol for 24 h, CCK-8 assays revealed no noticeable toxicity at concentrations below 100 μmol/L (Lv, 2024). Similarly, no significant differences were observed between rat chondrocytes treated with catalpol (at concentrations ranging from 0 to 1000 μmol/L) and untreated chondrocytes after 48 h, suggesting that catalpol does not exhibit toxicity to chondrocytes (Pang, 2023). These findings confirm that catalpol is safe for clinical use.

Recent advancements in biotechnology have led to the development of several novel dosage forms and administration routes for catalpol. Innovations include titanium-implanted catalpol, PLLA/gel-loaded catalpol, pyrazole heterocyclic modifications at the C10-position hydroxyl group of catalpol, catalpol hexapropionate (CP-6), catalpol lipid nanocarriers, catalpol freeze-dried powder injections (He, 2009), catalpol nasal drops, and catalpol gel, among others. A croton acylated catalpol derivative has been patented in China (CN 108912183 A), demonstrating excellent anti-aging properties and enhanced permeability across the blood-brain barrier, with esterification yields reaching 99.16%. These results provide compelling evidence for the improved targeting and retention of catalpol, showcasing promising potential for further research and development.

The TCM industry continues to develop new drugs to address critical, unmet therapeutic needs. Although the process of introducing a new monomeric TCM preparation to the market is time-consuming and costly, with 90% of drug candidates failing during clinical trials—a significant challenge in modern pharmaceutical development—recent progress has been made. For instance, a Phase IIa clinical trial of catalpol was conducted in Xining City, China. This study investigated the pharmacokinetics of catalpol tablets in patients with type 2 diabetes, assessing their effectiveness and safety at varying doses. The trial provided valuable insights into the clinical, pharmacological, and pharmacodynamic effects, as well as the distribution and metabolism of catalpol. The results helped determine the optimal dosage and therapeutic potential of catalpol, marking a breakthrough in the development of catalpol-based products. This progress is expected to accelerate the application and industrialization of catalpol-related products, strengthening the technological development of TCM.

**FIGURE 2:**
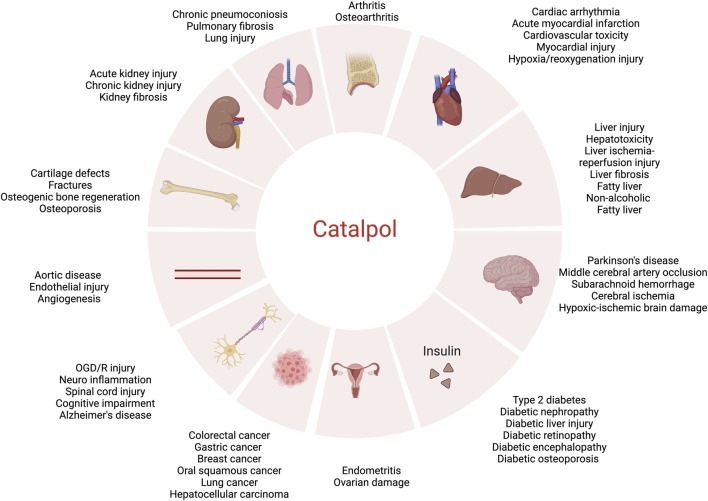
Pharmacological effects of catalpol.

While the pharmacological effects of catalpol have been extensively documented over the past decades, the majority of previous reports have been fragmented and lack a systematic overview. In recent years, catalpol has received extensive attention due to its safety and wide bioactivity. Although some reviews on catalpol have been published, most focused on specific diseases such as diabetes, cardiovascular diseases, Alzheimer’s diseases and so on, limited current studies such as the new dosage forms, and novel routes of administration of catalpol, which are key information for the application of catalpol. This is the first review to comprehensively summarize the pharmacological activity (Figure 2), safety, new dosage forms, and novel routes of administration of catalpol to provide ideas and references for its further in-depth research, development, and utilization.

## 2 Review methodology

To comprehensively review catalpol, a systematic search was conducted across major scientific databases including Medline, PubMed, ScienceDirect, China National Intellectual Property Administration, and CNKI, the range for the publication time is from 2020 to 2025. Moreover, a manual search was conducted to figure out pertinent articles. The literature retrieval process was designed to encompass a wide array of studies detailing both the therapeutic potential and development prospect of catalpol. The search strategy employed the use of specific keywords: “catalpol” “mechanisms of action” “Pharmacological effects” “safety” “new dosage forms” and “novel routes of administration” along with their relevant synonyms and related terms. The selection criteria for studies included in this review were predefined to include peer-reviewed research articles, patent documents and clinical trial reports published.

## 3 Pharmacological effects

### 3.1 Kidney diseases

Kidney is an organ with high oxygen consumption, which is rich in mitochondria and easy to produce ROS, which in turn leads to increased oxidative stress. At the same time, chronic inflammation is also an important driving factor for the progress of kidney disease. Lipopolysaccharide (LPS) induces acute kidney injury in rats, and catalpol mitigates this by suppressing the Toll-like receptor 4 (TLR4)/nuclear factor kappa-B (NF-κB) pathway, thereby reducing kidney tissue and glomerular endothelial damage while enhancing renal function (Hua et al., 2020). Catalpol also exerts protective effects against septic acute kidney injury by modulating Sirtuin 1 (SIRT1) and Nuclear Factor erythroid 2 (Nrf2)/Heme Oxygenase (HO-1) signaling pathways, synergistically promoting antioxidant and anti-inflammatory responses and alleviating sepsis-induced organ dysfunction in LPS-treated mice (Zhang and Qiang, 2023).

In a kidney injury model involving renal tubular epithelial cell line NRK-52E and mice exposed to aristolochic acid I, catalpol regulates the Nrf2/NF-κB pathway, significantly improving kidney anemia and fibrosis, preserving kidney structure and function, and providing renal protection (Liu Z. H. et al., 2024). Furthermore, catalpol mitigates Cisplatin-induced kidney injury and suppresses the inflammatory response, including reductions in Tumor Necrosis Factor-alpha (TNF-α), interleukin-6 (IL-6), IL-1β, IL-8, and iNOS, by activating Nrf2 and inhibiting the NF-κB signaling pathway (Zhang J. et al., 2020).

In a mouse model of chronic kidney disease induced by adenine, catalpol activates SIRT1 and inhibits NF-κB, thereby reducing inflammation, oxidative stress, and fibrosis, lowering kidney injury markers, and preventing DNA damage and apoptosis (Zaaba et al., 2023). In adriamycin-induced nephropathy in mice, catalpol reduces the levels of inflammatory cytokines in the kidney and alleviates kidney injury by upregulating SIRT1 and Multidrug Resistance-Associated Protein 2, while downregulating Transient Receptor Potential Cation Channel Subfamily C Member 6. Additionally, catalpol protects the podocyte cell line from adriamycin-induced damage by reducing adriamycin accumulation and intracellular free calcium, demonstrating potent renal protective effects (Zhang J. et al., 2019).

Catalpol exerts protective effects against Cisplatin-induced kidney injury through the mitochondrial-dependent pathway (Zhang J. et al., 2021). It also inhibits kidney fibrosis in unilateral ureteral obstruction rats by downregulating the wingless-type MMTV integration site family (Wnt)/β-catenin signaling pathway, significantly reducing the expression of Collagen I, Vimentin, and alpha-smooth muscle actin (α-SMA), while enhancing E-cadherin expression (Ruan, 2020). Catalpol alleviates Ang II-induced kidney injury in mice by inactivating the NF-κB and transforming growth factor-beta 1 (TGF-β1)/Smads signaling pathways (Cong et al., 2022). In a model of kidney injury induced by excessive fructose intake in mice, catalpol improved insulin sensitivity and hyperuricemia by inhibiting the activation of TLR4/myeloid differentiation primary response gene 88 (MyD88) signaling, ameliorating kidney inflammation, and protecting podocyte integrity (Chen Y. et al., 2024).

In 5/6 nephrectomy rats, catalpol downregulates the expression of TGF-β1 and connective tissue growth factor, inhibiting fibrous cell proliferation, reducing fibrotic tissue formation and inflammatory infiltration, and effectively blocking kidney fibrosis (Sun et al., 2020a). Using the same nephrectomy model, catalpol also alleviates micro-inflammation in rats with kidney fibrosis, a mechanism likely involving downregulation of abnormal adiponectin expression and inhibition of TNF-α and IL-6 release (Sun et al., 2020b).

**FIGURE 3:**
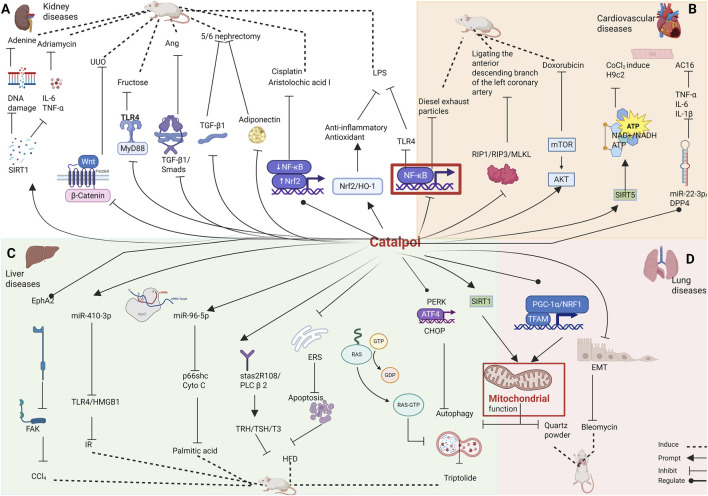
Effects of catalpol on the **(A)** kidney diseases, **(B)** cardiovascular diseases, **(C)** liver diseases, **(D)** lung diseases.

In summary, catalpol provides protective effects in kidney diseases. It inhibits kidney fibrosis and damage induced by LPS, aristolochic acid I, Cisplatin, adenine, adriamycin, Ang II, excessive fructose, unilateral ureteral obstruction, and 5/6 nephrectomy through its antioxidant and anti-inflammatory properties, as well as by modulating mitochondrial pathways. This evidence supports the potential use of catalpol in the disposal of kidney damage (Table 1; Figure 3).

**TABLE 1:** Pharmacological effects of catalpol in kidney diseases.

Pharmacology	Experimental animal and dose	Experimental cell and dose	Effect	Ref.
Kidney diseases	Intraperitoneal injection of LPS in rats 10 mg/kg	—	↓Scr ↓BUN ↓TNF-α ↓IL-6 ↓ET-1 ↓TLR4 ↓MyD88 ↓NF-κB p65 ↑NO	Hua et al. (2020)
	Adenine-induce rats 25, 50, 100 mg/kg	—	↓TNF-α ↓IL-1β ↓IL-6 ↓IL-8 ↓iNOS ↑IL-10 ↑Nrf2 ↑HO-1 ↑IκB ↓Keap1	Zhang J. et al. (2021)
	Adenine-induce mice 5 mg/kg	—	↓TNFα ↓IL-6 ↓cleaved caspase-3 ↑SIRT1 ↓NF-κB	Zaaba et al. (2023)
	Adriamycin induce mice 40, 80, 120 mg/kg	Adriamycin induce the mouse podocyte clone 5 cell line 100 μM	↑SOD ↓serum creatinine ↓BUN ↓malondialdehyde ↓IL-6 ↓TNF-α ↑MRP2 ↓TRPC6 ↑nephrin	Zhang J. et al. (2019)
	Unilateral ureteral obstruction in rats 3.2, 1.6, 0.8 g/kg	Human renal tubular epithelial cells 10, 50, 100 μg/mL	↓α-SMA ↓Vimentin ↓Collagen I ↑E-cadherin ↓Wnt5a ↓GSK-3β ↓β-catenin	Ruan (2020)
	Ang II-induce mice 25, 50, 100 mg/kg	Ang II-induce SV40 MES 13, NRK-49F, HK-2 1, 5, 10 µM	↓Collagen IV ↓TGF-β1 ↓TNF-α ↓IL-6	Cong et al. (2022)
	Rats were 5/6 Nephrectomised 10 mg/kg	—	↓TGF-β1 ↓CTGF	Sun et al. (2020a)
	Rats were 5/6 Nephrectomised 100, 50, 10 mg/kg	—	↓ADPN ↓TNF-α ↓IL-6	Sun et al. (2020b)

### 3.2 Cardiovascular diseases

Mixed lineage kinase domain-like protein (MLKL) is involved in the pathological process of cardiovascular diseases. Catalpol is effective against aconitine-induced cardiac arrhythmia in human-induced pluripotent stem cells (Yang L. et al., 2020). In a rat acute myocardial infarction model, created by ligating the anterior descending branch of the left coronary artery, catalpol inhibits myocardial cell necrosis and apoptosis by downregulating the receptor-interacting protein-1 (RIP1)/RIP3/MLKL pathway (Li H. S. et al., 2024).

Catalpol also prevents cardiovascular toxicity induced by acute exposure to diesel exhaust particles by inhibiting NF-κB activation (Nemmar et al., 2022). In the case of doxorubicin-induced cardiotoxicity, catalpol ameliorates the condition by reducing oxidative stress, myocardial apoptosis, and autophagy through activation of the protein kinase B (AKT)/mammalian target of rapamycin (mTOR) pathway (Liu B. et al., 2023). Additionally, catalpol reduces inflammation and oxidative stress in doxorubicin-treated H9c2 cardiomyoblasts by activating Peroxisome proliferator-activated receptor γ (PPAR-γ) (Jiang et al., 2020).

Catalpol regulates energy metabolism and inflammation by activating the SIRT5-mediated signaling pathway, providing protection in CoCl_2_-induced myocardial injury in H9c2 cells. This is achieved by increasing the nicotinamide adenine dinucleotide (NAD+/NADH) ratio and adenosine triphosphate (ATP) levels, while inhibiting lactic acid accumulation and reducing inflammatory factor release (Zheng Z. et al., 2024). In human cardiomyocyte AC16 cells subjected to hypoxia/reoxygenation (H/R) injury, catalpol regulates the miR-22-3p/Dipeptidyl peptidase-4 (DPP4) axis, promoting cell viability, reducing apoptosis (Li Z. et al., 2022).

Thus, catalpol provides protection in models of cardiac arrhythmia, acute myocardial infarction, cardiotoxicity, and myocardial injury. Its protective effects are primarily mediated through antioxidant and anti-inflammatory properties, as well as by modulating pathways such as RIP1/RIP3/MLKL, AKT/mTOR, and miR-22-3p/DPP4. This evidence offers a solid theoretical foundation for the future use of catalpol in the settlement of heart diseases (Table 2; Figure 3).

**TABLE 2:** Pharmacological effects of catalpol in cardiovascular diseases.

Pharmacology	Experimental animal and dose	Experimental cell and dose	Effect	Ref.
Cardiovascular diseases	—	Sconitine induce human-induced pluripotent stem cells 10, 100 µM	↓caspase-3 ↓caspase-9 ↓LDH	Yang L. et al. (2020)
	Ligating the anterior descending branch of the left coronary artery induce rat 30, 60 mg/kg	—	↓TNF-α ↓IL-1β ↓IL-6 ↓RIP1 ↓RIP3 ↓MLKL	Liu B. et al. (2023)
	Diesel exhaust particles induce mice 5 mg/kg	—	↓C-reactive proteins ↓fibrinogen ↓PAI-1 ↓PAI-P ↓E-selectins ↑SOD ↓NO ↓TNF-α ↓IL1-β ↓IL-6 ↓DNA damage ↓Phopho-IκBα	Jiang and Zhang. (2020)
	—	CoCl2 induce H9c2 cells 2.5, 5, 10, 20, 40, 80 μmol/L	↑NAD+/NADH ↑ATP ↓lactic acid ↓TNF-α ↓IL-1β ↓IL-6	Zheng et al. (2024a)

### 3.3 Liver diseases

Liver disease has a long course and complicated pathogenesis. Fatty acid accumulation, inflammatory cells and apoptosis are all inextricably linked with it. A mouse model of cholestasis was established by bile duct ligation, and catalpol was shown to reduce liver injury in this model by inhibiting oxidative stress, enhancing mitochondrial membrane potential, and increasing ATP and glutathione content (Gao et al., 2020). Catalpol also downregulates the activating the Janus kinase (JAK)/signal transducer and activator of transcription (STAT) signaling pathway to mitigate liver injury induced by heat stroke (Ji et al., 2024). In both *in vitro* SIRT1 knockout/overexpression (AML12 cells) and *in vivo* liver-specific SIRT1 knockout models, catalpol alleviates Triptolide-induced liver injury by activating SIRT1, improving mitochondrial dysfunction, and reducing glucose metabolism disorders and oxidative stress (Nie et al., 2024). Additionally, catalpol protects against Triptolide-induced hepatotoxicity by inhibiting excessive autophagy via the protein kinase RNA-like endoplasmic reticulum kinase (PERK)/Activating Transcription Factor 4 (ATF4)/CHOP pathway, reversing liver function indices, autophagy levels, and apoptotic protein expression (Zhang L. et al., 2022). In the rat liver ischemia-reperfusion injury model and hepatocyte HL-7702 experiments, catalpol upregulated miR-410-3p, inhibiting the activation of the TLR4/High Mobility Group Box 1 (HMGB1) signaling pathway, alleviating aseptic inflammation in rat liver tissue, and inhibiting oxidation, thus providing protection against ischemia-reperfusion injury (Wang, 2023; Feng et al., 2023).

In a CCl_4_-induced liver fibrosis model, catalpol inhibits autophagy in hepatocytes by reducing Rac1-GTP, which in turn inhibits hepatic stellate cell activation. This effect occurs not only by reducing the formation of hepatocyte-derived extracellular vesicles but also by altering their contents, thereby attenuating liver fibrosis (Xie et al., 2023). *In vivo*, CCl_4_-induced liver fibrosis in mice and TGF-β-stimulated LX-2 cells were used to model fibrosis *in vitro*. Catalpol directly targets Ephrin type-A receptor 2 (EphA2) to reduce its binding with focal adhesion kinases (FAK), significantly inhibiting the FAK/Src pathway. This inhibition suppresses aerobic glycolysis in activated hepatic stellate cells, resulting in reduced liver injury, fibrogenesis, and inflammation in mice (Zhang Q. et al., 2024).

In experiments involving mice induced by an HFD and 293T cells cultured *in vitro*, catalpol activates the hypothalamic Bitter Taste Receptors (stas2R108)/PLC β2 pathway, promoting the secretion of thyrotropin-releasing hormone (TRH), thyroid-stimulating hormone (TSH), and triiodothyronine (T3). This leads to the improvement of lipid vacuolation and lipid droplet accumulation in the liver, a reduction in the size of white adipocytes, and a decrease in serum free fatty acid content (Xu, 2024). Catalpol inhibits lipid accumulation, apoptosis, and oxidative stress in the HepG2 cell model induced by palmitic acid. It does so by increasing the levels of miR-96-5p and decreasing the expression of p66shc and cytochrome c (Cyto C) (Xu, 2020; Zhang Y. et al., 2020). In models of non-alcoholic fatty liver induced by a high-fat diet in mice and lipotoxicity induced by palmitate in human hepatocellular carcinoma HepG2 cells, catalpol inhibits hepatocyte apoptosis by relieving endoplasmic reticulum stress, thereby protecting against liver injury (Tian et al., 2021; Tian, 2020; Tian et al., 2020).

Catalpol shows promising results as a potential therapy for liver system diseases, including liver injury, liver fibrosis, and fatty liver. This compound could be developed into a therapeutic drug for liver diseases (Table 3; Figure 3).

**TABLE 3:** Pharmacological effects of catalpol in liver diseases.

Pharmacology	Experimental animal and dose	Experimental cell and dose	Effect	Ref.
Liver diseases	Bile duct ligation induce mice 5, 10 mg/kg	—	↓TNF-α ↓IL-1β ↓IL-6 ↓ROS ↑ATP ↑GSH ↓MDA ↓Cytochrome c ↑Bcl-2 ↓Bax ↓Cleaved caspase-3	Zhang M. F. et al. (2022)
	Triptolide induce mice 1.5, 3, 4.5 mg/kg	Triptolide induce AML12 cells 100–400 nM	↓ALT ↓AST ↓LDH ↓MDA ↓4-hydroxynonenal ↑mtDNA copy number	Nie et al. (2024)
	Rat liver was ischemic for 1 h and perfused for 6 h 5, 25, 50 mg/kg	Hepatocyte HL-7702 20, 40, 80 μM	↓AST ↓ALT ↑SOD ↑GSH ↓MDA ↓IL-1β ↓TNF-α ↑miR-410-3p ↓HMGB1 ↓TLR-4 ↓MyD88 ↓NF-κB ↑IκB-α	Wang (2023); Feng et al. (2023)
	CCl_4_ induce mice 10 mg/kg	—	↓ACTA2 ↓Col1a1 ↓α-SMA ↓Rac1-GTP ↓LC3-II ↓SQSTM1	Zhang Q. et al. (2022)
	High-fat induced mice 5, 10, 20, 40, 80 mg/kg	293T cells induced by high fat 1, 10, 100 μM	↑HDL-C ↓TG ↓TC ↓ LDL-C ↓AST ↓ALT ↓Free fatty acids ↑PPAR-α ↑CPT-1α ↓ACC1 ↓FASN	Xu (2024)
	—	Palmitic acid induces HepG2 cells 5, 20, 80 μM	↓P66Shc ↓Cyto C ↓Apoptosis	Xu (2020); Zhang B. et al. (2020)
	Mice induced by high-fat diet 100, 200, 400 mg/kg	Palmitic acid induces human hepatocellular carcinoma cell line HepG2 100, 200, 400 μmol/L	↓TG ↓TC ↓ALT ↓AST ↓p-PERK ↓BiP ↓IRE1α ↓ATF6 ↓CHOP ↓p-JNK ↓Caspase-12 ↓Caspase-9 ↓Caspase-3	Tian et al. (2021); Tian (2020); Tian et al. (2020)

### 3.4 Lung diseases

At present, most of the treatment of lung diseases lacks specific drugs, it is very important to find suitable and safe drugs for lung disease. Catalpol mitigates liver and lung injury in rats with common bile duct ligation by regulating bile acid overexpression, enhancing TGR5 expression, and reducing Farnesoid X Receptor (FXR) levels, thereby offering protection against lung injury in hepatopulmonary syndrome (Zeng et al., 2024). In a rat model of chronic pneumoconiosis induced by intratracheal injection of quartz powder, catalpol promotes mitochondrial biogenesis through the peroxisome proliferator-activated receptor-γ coactivator-1 alpha (PGC-1α)/nuclear respiratory factor 1 (NRF1) and Transcription Factor A (TFAM) pathways, enhancing mitochondrial function in skeletal muscle, reducing muscle atrophy, and improving exercise capacity (Liu W. et al., 2022). Catalpol simultaneously blocks the Angiotensin II (Ang II) and TGF-β pathways to attenuate pulmonary fibrosis in bleomycin-induced pulmonary fibrosis in mice. It reduces inflammation, alleviates collagen deposition, and mitigates epithelial-mesenchymal transition (EMT) (Yu et al., 2022; Yang F. et al., 2021).

These *in vivo* experiments demonstrate that catalpol can protect against lung diseases caused by bile duct ligation, quartz powder, and bleomycin. However, its efficacy in treating lung diseases *in vitro* and clinical settings requires validation through large-scale, standardized studies to support its broader application in lung disease therapies (Table 4; Figure 3).

**TABLE 4:** Pharmacological effects of catalpol in lung diseases.

Pharmacology	Experimental animal and dose	Experimental cell and dose	Effect	Ref.
Lung diseases	Common bile duct ligation induced rats 5.0 mg/kg	—	↓ALT ↓AST ↓Gamma-glutamyltransferase ↓Total bile acid ↓Total Bilirubin ↑TGR5 ↓FXR	Zeng et al. (2024)
	Intratracheal injection of quartz dust induced rats 100, 50 mg/kg	—	↑ATP ↑Mitochondrial membrane potential ↑SDH ↑SOD ↓MDA ↑Pgc-1α ↑Nrf1 ↑Tfam	Liu W. et al. (2022)
	Bleomycin induces mice 200, 100 mg/kg	—	↓MMP2 ↓MMP9 ↑E-cadherin ↓N-Cadherin ↓α-SMA ↓Ang II ↓TGF-β1 ↓phospho-Smad2	Yu et al. (2022); Yang F. et al. (2021)

### 3.5 Diabetes

Chemical drugs are effective in the treatment of diabetes, but the drug has a single target. Long-term use is likely to lead to ketoacidosis, secondary failure, chronic liver and kidney damage and gastrointestinal indigestion, so it is urgent to find more suitable and safe hypoglycemic drugs. As early as 2017, China Food and Drug Administration approved “catalpol tablets” as a clinical trial of traditional Chinese medicine to treat diabetes. *In vivo*, type 2 diabetes was induced in mice through a high-fat diet (HFD) combined with streptozotocin (STZ) injection. *In vitro*, insulin resistance was induced in HepG2 cells by glucosamine administration. Catalpol enhances the expression of adenosine 5′-monophosphate (AMP)-activated protein kinase (AMPK) in the skeletal muscle of diabetic rats, improving glucose metabolism, maintaining glucose homeostasis, and enhancing insulin sensitivity (Shen, 2023; Xu D. Q. et al., 2020; Xu et al., 2020b). Catalpol reduces blood glucose levels in STZ-induced diabetic rats through activation of the PGC-1α signaling pathway (Huang X. X. et al., 2022). It also activates the AMPK/SIRT1/PGC-1α/PPAR-γ pathway in the skeletal muscle of type 2 diabetic mice, leading to significant improvements in insulin sensitivity and mitochondrial respiration (Yap et al., 2020; Li Y. et al., 2024). Additionally, catalpol improves insulin resistance and lipid metabolism disorders in diabetic mice induced by HFD and STZ by inhibiting miR-101-3p and upregulating Fos-related antigen 2 (Xu C. F. et al., 2024). In a study of STZ-damaged INS-1 cells, catalpol alleviates ERS, reduces oxidative stress, protects β-cell function, and enhances insulin synthesis and secretion. Similarly, catalpol improves glucose consumption in the insulin-resistant state of IR-HepG2 cells induced by glucosamine, enhances hepatocyte glycogen synthesis, improves glucose uptake, and upregulates Adiponectin and O-GlcNAc transferase (Ding, 2023; Elhassan et al., 2021). In experiments with H_2_O_2_-induced rat insulinoma INS-1 cells and high-glucose-induced EA.hy926 cells, catalpol activates the Nrf2/HO-1 antioxidant signaling pathway, inhibits reactive oxygen species (ROS) production, reduces oxidative damage and β-cell apoptosis, and promotes insulin synthesis and secretion (Xiao et al., 2022; Xiao, 2021; Zhu Q. W. et al., 2021).

Catalpol also alleviates oxidative stress in diabetic rats, potentially through inhibition of oxidized low-density lipoprotein (oxLDL)/LDL and NF-κB signaling pathways, thereby mitigating macroangiopathy induced by a high-fat and high-sugar diet combined with STZ in type 2 diabetic rats (Luo et al., 2023). Furthermore, catalpol significantly reduces DNA damage in EA.hy926 cells induced by high glucose by increases the expression of autophagy-related proteins beclin-1 and LC3-II/LC3-I (Li W. T. et al., 2022).

Catalpol exerts a protective effect on the kidneys of diabetic rats (Zhang C. et al., 2022). High glucose stimulates podocytes in mice, and catalpol inhibits podocyte pyroptosis and reduces inflammation by blocking the ROS/pyrin domain containing protein (NLR) family, NLRP3/caspase-1 pathway (Chen H. et al., 2023). In a type 2 diabetic mouse model established by HFD combined with STZ, catalpol improves glucose metabolism, attenuates renal inflammation, and preserves renal structure and function by inhibiting the TGF-β1/Smad3 signaling pathway (Zhang H. W. et al., 2024) and activating the AMPK/SIRT1/NF-κB pathway (Chen J. et al., 2020). This combined action delays the progression of diabetic nephropathy. In a STZ-induced diabetic nephropathy rat model, catalpol maintains endoplasmic reticulum homeostasis by downregulating the expression of PERK and CHOP in renal tissue, thereby alleviating renal injury associated with diabetic nephropathy (Guo et al., 2024). In a diabetic nephropathy mouse model overexpressing the receptor for advanced glycation end products (RAGE) and an AGE-induced endothelial-macrophage co-culture injury model, catalpol regulates the RAGE/Ras homolog gene family member A (RhoA)/Rho-associated kinase (ROCK) signaling pathway, interferes with the interaction between macrophages and endothelial cells, reduces endothelial injury and chemokine secretion, inhibits macrophage migration, and restores the M1/M2 phenotypic balance, improving the pathological damage in diabetic nephropathy (Shu, 2021; Shu et al., 2020; Shu et al., 2021).

Catalpol also promotes the uncoupling of Galectin-3 and CD146 molecular complexes induced by AGEs, improving endothelial damage in hepatic sinusoids and reducing the release of monocyte chemoattractant protein-1 (MCP-1) and intercellular adhesion molecule-1 (ICAM-1). This leads to reduced macrophage activation and nitric oxide (NO) secretion, offering protective effects to the hepatic sinusoidal endothelium. *In vitro* experiments have provided preliminary evidence for its mechanism in mitigating diabetic liver injury (Sun et al., 2023a; Sun et al., 2023b). Furthermore, catalpol inhibits inflammatory responses through the PPARγ/NF-κB signaling pathway, alleviating liver injury induced by a high-fat and high-sugar diet combined with STZ in type 2 diabetic rats (Wang R. R. et al., 2022).

Catalpol regulates glucose metabolism in high-sugar and high-fat-induced type 2 diabetic mice by modulating bile acid levels in the liver. It improves liver and retinal damage, significantly increases the levels of vitamin B12 and folic acid in the liver, and reduces the risk of peripheral neuropathy (Zeng et al., 2022). Additionally, catalpol regulates inflammatory factors by inhibiting the AGE/RAGE/NF-κB signaling pathway, restoring retinal adhesion protein in KK-Ay diabetic mice, improving retinal vascular permeability, and offering potential prevention and computing for diabetic retinopathy (Wu and Du, 2021).

Catalpol reduces apoptosis in SH-SY5Y cells induced by high glucose by upregulating the levels of B-cell lymphoma/leukemia-2 (Bcl-2) protein and Yes-associated protein (YAP), providing a research foundation for the study of diabetic encephalopathy (Han et al., 2021). It also enhances glycolysis via the AGEs/RAGE signaling pathway, activating key rate-limiting enzyme to significantly improves testicular lesions in KK-Ay spontaneously diabetic mice fed an HFD (Chen Y. P. et al., 2023; Chen Y. P. et al., 2020; Zhu Y. et al., 2021).

In a diabetic rat model induced by a high-fat and high-sugar diet combined with STZ, catalpol inhibits the release of inflammatory factors by downregulating the NLRP3/Caspase-1 signaling pathway related to pyroptosis. This results in a significant reduction in myocardial injury and an improvement in cardiac function in diabetic cardiomyopathy rats. In STZ-induced rats with left anterior descending coronary artery ligation, catalpol exerts cardioprotective effects by reducing inflammation and alleviating ERS (Yu et al., 2024; Bi et al., 2020; Xu R. et al., 2024). Catalpol also demonstrates a protective effect against diabetic osteoporosis (Cheng J. et al., 2020). In a diabetic osteoporosis mouse model, catalpol regulates the differentiation and migration of osteoblasts, improving bone formation markers. Additionally, it enhances cell motility and scattering following gap formation in high glucose-induced MC3T3-E1 cells (Zhao et al., 2021).

**FIGURE 4:**
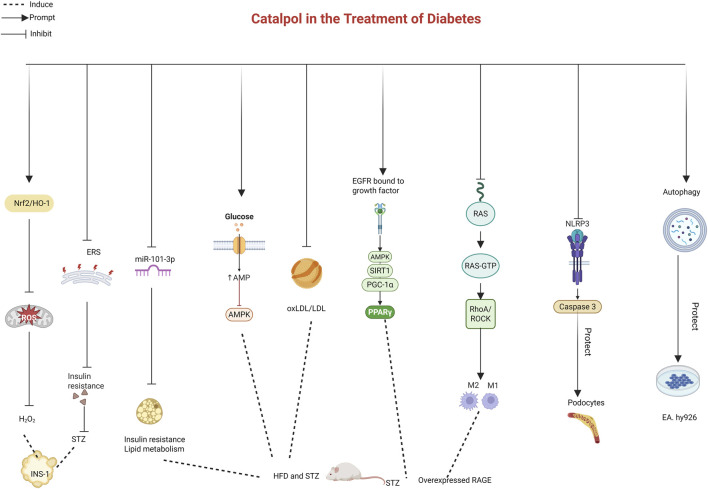
Effects of catalpol on the diabetes.

Taken together, these studies indicate that catalpol is effective in treating diabetes and its complications, including diabetic nephropathy, diabetic angiopathy, diabetic liver injury, diabetic myocardial injury, diabetic testicular lesions, and diabetic osteoporosis. Catalpol exerts its therapeutic effects through multiple pathways, including relieving ERS, inhibiting oxidative stress, suppressing inflammation, promoting autophagy, and inhibiting pyroptosis. Future research utilizing metagenomics and transcriptomics could further elucidate catalpol’s role in diabetes and clarify the mechanisms underlying metabolic changes (Table 5; Figure 4).

**TABLE 5:** Pharmacological effects of catalpol in diabetes.

Pharmacology	Experimental animal and dose	Experimental cell and dose	Effect	Ref.
Diabetes	Rats induced by high fat and high sugar combined with STZ 2.5, 5, 10 m g/kg	—	↑AMPK ↑GLUT4	Shen (2023); Xu et al. (2020a); Xu et al. (2020b)
	Rats were injected with STZ 25, 50, 100 mg/kg	—	↓TC ↓TG ↑PGC1α ↑AMPK ↑SIRT1	Huang X. X. et al. (2022); Yap et al. (2020)
	—	STZS induces INS-1 cells Glucosamine induces IR-HepG2 cells 0.05, 0.2, 1, 5, 12.5, 25, 50 μmol/L	↓Bax ↑Bcl-2 ↑Calcium ion ↓IRE1α ↑SOD ↓MDA ↑CAT ↑Nrf2 ↑Foxo1 ↑Pancreatic and duodenal homeobox 1/Insulin promoter factor l ↑Glut2 ↑Pancreatic and duodenal homeobox 1/Insulin promoter factor l ↑Adiponectin ↑O-GlcNAc transferase	Ding (2023); Elhassan et al. (2021)
	—	H_2_O_2_ induces INS-1 cells 1, 5, 10, 20, 40, 80, 160 μmol/L	↓ROS ↓MDA ↑SOD ↑Nrf2 ↑Keap1 ↑ERK ↑HO-1 ↑pancreatic-duodenal homeobox factor-1 ↑GLUT2	Xiao et al. (2022); Xiao (2021); Zhu Q. W. et al. (2021)
	Rats induced by high fat and high sugar combined with STZ 10 mg/kg	—	↓FBG ↓TC ↓TG ↓LDL ↓MCP⁃1 ↓oxLDL ↓LOX⁃1 ↓NF⁃κB ↓p65 ↓MCP⁃1	Luo et al. (2023)
	—	High glucose induces EA. hy926 cells 0.5, 0.05, 0.5 mmol/L	↓8-hydroxy-2′-deoxyguanosine ↓ROS ↓γH2AX ↑beclin-1 ↑LC3-II/LC3-I ↓p62	Li W. T. et al. (2022)
	—	High glucose induces mouse podocytes 2, 5, 10 μmol/L	↓ROS ↓IL-1β ↓IL-18 ↓NLRP3 ↓Active-caspase1 ↓GSDMD - N	Chen H. et al. (2023)
	Mice induced by high-fat diet combined with low-dose STZ 100 mg/kg	—	↓Oral glucose tolerance test ↓Total cholesterol ↓TG ↓SCr ↓BUN ↓Fasting insulin ↓Insulin resistance index ↑Insulin sensitivity index ↓IL-1 ↓TGF-β1 ↓Smad3 ↓IL-6 ↓TNF-α ↓COL-III ↑MMP9	Zhang Y. Z. (2024); Chen J. et al. (2020)
	STZ induced rats 50, 100, 150 mg/kg	—	↓PERK ↓CHOP	Guo et al. (2024)
	KK/Ay mice induced by high fat and high glucose 50, 1 00 mg/kg	Glomerular endothelial cells and macrophages 50, 100, 200, 400 mg/L	↓MCP-1 ↓M-CSF ↓IL-12 ↑IL-10 ↑Occlidin ↑VE-cadherin ↓iNOS ↑Arg-1	Shu (2024); Shu et al. (2020); Shu et al. (2021)
	—	Rat liver sinusoidal endothelial cells 0, 0.1, 1, 10 μmol/L	↓LDH ↓MCP-1 ↓ICAM-1	Sun et al. (2023a); Sun et al. (2023)
	Rats induced by high fat and high sugar combined with STZ 10, 50, 100 mg/kg	—	↓TC ↓TG ↓ALT ↓AST ↓Glycatedhemoglo bin ↓IL-6 ↓TNF-α ↑IκB ↑PPARγ ↓NF-κB	Wang J. et al. (2022)
	High glucose and high fat induced db/db mice 150, 200, 250 mg/kg	—	↓Alanine aminotransferase ↓Glutamic oxaloacetic aminotransferase ↓Total bilirubin ↓Albumin ↓Direct bilirubin ↓Indirect bilirubin ↓Ursodeoxycholic acid ↑Lithocholic acid ↑Deoxycholic acid ↑Chenodeoxycholic acid ↑Folic acid ↑Vitamin B12	Zeng et al. (2022)
	KK-ay diabetic mice 50, 100 mg/kg	—	↓IL-1β ↓TNF-α ↓VEGF ↓AGE ↑VE-cadherin ↓RAGE ↓P-NF-κB p65	Wu and Du (2021)
	—	High glucose induces human neuroblastoma SH-SY5Y cells 0.5, 1, 2 mg/mL	↓ROS ↑YAP ↑Bcl-2	Han et al. (2021)
	High-fat-induced KK-Ay spontaneous diabetic mice 100 mg/kg	—	↑GSH ↑SOD ↑LDH ↑Metabolic product glucose fructose-1 ↑6-diphosphate ↑3-phosphate glycerate ↑3-phosphate glyceraldehyde ↑lactic acid and pyruvate ↑Hexokinase ↑Phosphofructose kinase ↑Pyruvate kinase ↑LDH ↓AGEs ↓RAGE ↓Bax/Bcl-2	Chen H. et al. (2023); Chen T. P. et al. (2020); Zhu Q. W. et al. (2021)
	Rats induced by high fat and high sugar combined with STZ 100, 300, 900 mg/kg	—	↓NLRP3 ↓Caspase-1 ↓IL-1β ↓IL-18	Yu et al. (2024); Bi et al. (2020)
	High-fat diet and intraperitoneal STZ induce mice 30, 90 mg/kg	High glucose induced MC3T3-E1 cells 1, 10 μM	↑ALP ↑Osteocalcin ↓RANKL ↑OPG/RANKL	Cheng J. et al. (2020); Zhao et al. (2021)

### 3.6 Arthritis

Arthritis is an autoimmune disease, and its pathogenesis may be closely related to the abnormal expression of inflammatory factors and the abnormal activation of immune cells. Rats were injected with 4% papain into the left knee joint to replicate a rat model of knee osteoarthritis. Catalpol delays the progression of knee osteoarthritis by reducing the protein levels of IL-1β, Galectin-3, and S100A12 in the synovial tissue of knee osteoarthritis rats (He et al., 2020a; He et al., 2020b; Zhang B. et al., 2020). Additionally, catalpol inhibits the activation of the nucleotide-binding oligomerization domain containing 2 (NOD2)/NF-κB/mitogen-activated protein kinase (MAPK) signaling pathway, playing an anti-inflammatory and protective role in mouse chondrocytes. It effectively slows the disease process in a mouse osteoarthritis model induced by the improved Hulth method, significantly reducing the protein level of NOD2 in mouse cartilage tissue (Pang, 2023), thereby alleviating the inflammatory damage in cartilage tissue associated with osteoarthritis (Wu et al., 2025). In a model of human rheumatoid arthritis fibroblasts (HFLS-RA) induced by TNF-α and a rat model of rheumatoid arthritis established by type II collagen injection, catalpol decreases the production levels of IL-6, MCP-1, and IL-1β by weakening the signaling activity of YAP/transcriptional co-activator with PDZ-binding motif (TAZ). It also inhibits excessive cell proliferation, promotes cell apoptosis (Jiang et al., 2024).

In experiments with naïve CD4^+^ T cells isolated from the spleen of C57BL/6 mice, catalpol downregulates the levels of glycolysis products pyruvate and lactate by suppressing the expression of pyruvate kinase M2 (PKM2) and LDHA. This interference with glycolysis disrupts Th17 cell differentiation, thereby alleviating rheumatoid arthritis (Ge et al., 2024). Additionally, in CD4^+^ T cells from the peripheral blood of patients with rheumatoid arthritis, catalpol upregulates miR-143-3p, inhibits the abnormal differentiation of Th17 cells, and downregulates glycolysis, thereby regulating the immune balance and exerting anti-inflammatory effects (Shen et al., 2022; Di et al., 2022).

In IL-1β-induced inflammation in human knee chondrocytes, catalpol reduces the release of inflammatory factors, while inhibiting apoptosis by upregulating miR-140-5p (Ma et al., 2023; Pang et al., 2023). In ATDC5 chondrocytes derived from mouse teratocarcinoma cells and stimulated with IL-1β to simulate the osteoarthritis cellular environment, catalpol significantly reduces matrix metalloproteinases (MMP-1, -3, -13) and a disintegrin and metalloproteinase with thrombospondin motifs (ADAMTS-4, -5), demonstrating anti-cartilage degradation activity (Cai et al., 2024; Yin et al., 2024).

When adriamycin induces aging in ATDC5 chondrocytes, catalpol delays the progression of knee osteoarthritis by promoting apoptosis and reducing aging-related phenotypes such as P21 and P53, as well as MMP13 and IL-6 (Jia et al., 2024).

**FIGURE 5:**
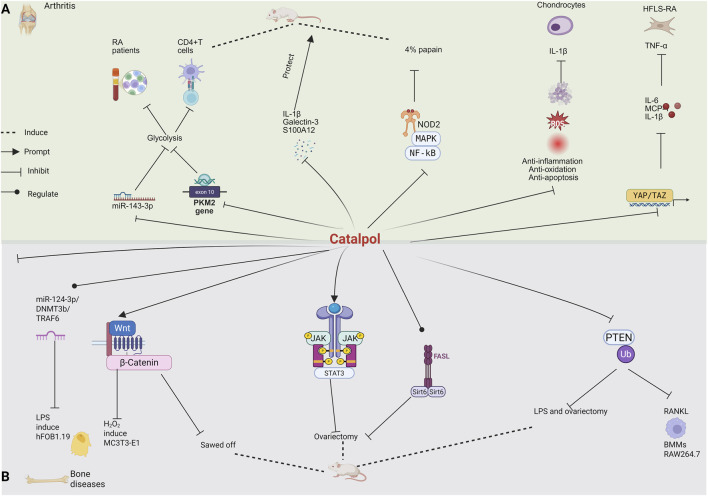
Effects of catalpol on the **(A)** arthritis and **(B)** bone diseases.

In summary, catalpol effectively inhibits inflammation, oxidative stress, and apoptosis, influencing multiple related signaling pathways to treat arthritis. It also acts on immune cells such as CD4^+^ T and Th17 cells, positioning it as a potential natural anti-inflammatory therapy for arthritis. The results from human, animal, and cell-based experiments support catalpol’s efficacy as a treatment for arthritis, confirming its therapeutic potential (Table 6; Figure 5).

**TABLE 6:** Pharmacological effects of catalpol in arthritis.

Pharmacology	Experimental animal and dose	Experimental cell and dose	Effect	Ref.
Arthritis	4% papain induced rats 100, 10 mg/kg	—	↓S100A12 ↓IL-1β ↓Galectin-3	He et al. (2020a); He et al. (2020b); Zhang B. et al. (2020)
	Construction of osteoarthritis mice by improved hulth method 100 mg/kg	IL-1β induces mouse chondrocytes 0, 0.1, 1, 10, 100, 1000 μmol/L	↓TNF-α ↓IL-1 ↓IL-6 ↓IL-12 ↓COX-2 ↓iNOS ↓MMP3 ↓MMP13 ↓NOD2 ↑p65 ↓IKBα ↓ERK ↓JNK	Pang (2023)
	Rats were injected with type II collagen 20.8 mg/kg	TNF-α induces HFLS-RA cells 20 μg/mL	↓IL-6 ↓MCP-1 ↓IL-1β ↓Cyclin D1 ↓YAP ↓TAZ	Jiang et al. (2024)
	NaiveCD4+T cells isolated from the spleen of C57BL/6 mice 20, 40, 80 μg/mL	—	↓PKM2 ↓LDHA ↓RORγt ↓STAT3	Ge et al. (2024)
	IL-1β induces human knee chondrocytes 10, 20, 50 ng/L	—	↓Apoptosis rate ↓Cleaved-caspase 3 ↓Cleaved- caspase 9 ↑miR-140-5p ↓IL-6 ↓TNF-α ↓IFN-γ	Cai et al. (2024)
	Rats were injected with incomplete freundsadjuvant chicken type II colagen 15, 30 mg/kg	—	↓L-1β ↓IL-6 ↓TNF-α ↓CTX-I ↓CTX- II ↓Cleaved-caspase-3 ↓MMP-3 ↓MMP-13 ↓NLRP3 ↑AMPK	Yin et al. (2024)
	—	Adriamycin induces ATDC5 chondrocytes 1, 80 μmol/L	↓P21 ↓P53 ↓MMP13 ↓IL-6 ↑Type II collagen	Jia et al. (2024)

### 3.7 Bone diseases

Osteoblast is the main functional cell leading bone formation in bone metabolism, which is very important for bone growth and bone balance maintenance. Osteoblast differentiation is regulated by many signaling pathways, and abnormal activation or inhibition of these pathways may lead to imbalance of bone formation, and catalpol can promote bone effect by targeting key molecules regulating these signaling pathways (Stegen and Carmeliet, 2024). Catalpol holds potential for the disposal of joint diseases (Chen et al., 2022). In a mouse model with full-thickness articular cartilage defects created on the trochlear groove using a 26G needle, catalpol contributes to cartilage regeneration, bone structure improvement, and increased matrix anabolism by stimulating endogenous mesenchymal stem cells (MSCs) in the cartilage, thus promoting the repair of localized cartilage defects (Wu et al., 2024a). In another experiment, where the middle femur of rats was sawed off by a wire saw, catalpol upregulates the expression of bone morphogenetic protein 2 (BMP-2), activates the Wnt/β-catenin signaling pathway, and promotes femoral fracture healing in rats (Cao et al., 2021). Catalpol also inhibits oxidative stress and inflammation in MC3T3-E1 cells induced by H_2_O_2_ by downregulating forkhead box O3 (FoxO3), activating the Wnt/β-catenin signaling pathway, thereby improving cell viability, osteogenic differentiation, and inhibiting apoptosis (Duan et al., 2024).

Furthermore, catalpol can inhibit the ubiquitination and degradation of PTEN, block the NF-κB and AKT signaling pathways, and prevent the differentiation of bone marrow-derived macrophages (BMMs) and RAW264.7 cells induced by receptor activator of nuclear factor kappa-B ligand (RANKL) into osteoclasts. It improves bone loss induced by LPS and ovariectomy by inhibiting osteoclast activity (Meng et al., 2020; Meng, 2020). In LPS-induced hFOB1.19 cells, catalpol antagonizes inflammation and suppresses osteoblast differentiation via regulating the miR-124-3p/DNA (cytosine-5)-methyltransferase 3B (DNMT3b)/TNF receptor-associated factor 6 (TRAF6) axis (Zhang P. et al., 2024). Catalpol also promotes osteogenic bone regeneration and vessel formation in bone marrow-derived stem cells (BMSC) and ovariectomy-induced osteoporosis calvarial defect rat models by activating the JAK2/STAT3 axis (Chen L. et al., 2021). In a female rat model of postmenopausal osteoporosis induced by ovariectomy, catalpol promotes osteoclast apoptosis via the NAD-dependent protein deacetylase SIRT6/Estrogen Receptor Alpha (Erα)/Fas Ligand (FasL) axis, thus preventing estrogen deficiency-induced osteoporosis (Chen S. et al., 2024). Additionally, catalpol enhances human periodontal ligament stem cell proliferation and promotes periodontal tissue remodeling in rat orthodontic tooth movement models (Hu J. et al., 2024). In glucocorticoid-induced osteoporosis mice, catalpol upregulates polycystic kidney disease-1 protein expression, reduces oxidative stress, promotes the mRNA expression of bone formation markers, and restores bone microarchitecture while increasing bone mass (Xu et al., 2023).

Currently, bone diseases are primarily managed with antibiotics. However, excessive and prolonged use of this approach has led to antibiotic resistance. Thus, the identification of new therapies for managing bone diseases is essential. The studies above confirm that catalpol reduces osteoclast activity and improves osteogenic differentiation in bone diseases primarily through pathways such as Wnt/β-catenin, JAK2/STAT3, miR-124-3p/DNMT3b/TRAF6, and Sirt6/Erα/FasL (Table 7; Figure 5).

**TABLE 7:** Pharmacological effects of catalpol in bone diseases.

Pharmacology	Experimental animal and dose	Experimental cell and dose	Effect	Ref.
Bone diseases	Full-thickness articular cartilage defect was created on the trochlear groove using 26 G needle in mice 10, 30 mg/kg	C3H10 T1/2 cells 0–1600 μM	↑Indicator of matrix anabolism and hyaline cartilage: Col2 ↑Sox9 ↑Col2 ↑Aggrecan genes ↑Cartilage matrix synthesis and accumulation ↑CD90	Duan et al. (2024)
	Saw the middle femur of rats with a wire saw 50 mg/kg	—	↑ALP ↑BMP-2 ↑OCN ↑Runx2 ↑ColI ↑β-catenin ↑cyclinD1 ↑c-myc	Meng et al. (2020)
	—	H_2_O_2_ induces mouse osteoblasts MC3T3-E1 100 μmol/L	↓ROS ↑CAT ↑SOD ↓IL-6 ↓IL-1β	Meng (2020)
	LPS and ovariectomy induced mice 10, 30 mg/kg	RANKL induces macrophages (BMMs) and RAW264.7 cells 100, 200, 400 uM	Osteoclast differentiation gene ↓CTSK ↓TRAP ↓CTR ↓DC-STAMP ↓V-ATPase d2 ↓NFATc1 ↓F-actin ↓CTX-1	Zhang M. F. et al. (2024); Chen et al. (2021a); Chen et al. (2024b)
	Ovariectomy induced rat 5, 10, 20 mg/kg	—	Osteoclast apoptosis-related proteins ↑Sirt6 ↑Erα ↑FasL ↑NFATc1 ↑cleaved-caspase 8 ↑cleaved-caspase 3 ↑Bax ↓NFATc1 ↓Ctsk ↓Oscar ↓Trap	Xu et al. (2023)

### 3.8 Brain diseases

At present, western medicine used to treat brain diseases is only symptomatic treatment, with a single approach and limited curative effect. In recent years, with the deepening of the research on brain diseases, the exploration and application of catalpol in the therapy of brain diseases have gradually increased. Catalpol demonstrates a neuroprotective effect in a 1-methyl-4-phenyl-1,2,3,6-tetrahydropyridine (MPTP)-induced Parkinson’s disease model in mice. By blocking the MKK4/JNK signaling pathway, catalpol inhibits the activation of ROS and caspase proteins, and increases the expression of tyrosine hydroxylase in the substantia nigra and striatum of mice (Tang et al., 2024). It also inhibits MPTP-induced oxidative stress in the substantia nigra of subacute Parkinson’s mice by regulating the Nrf2/HO-1/NAD(P)H:quinone oxidoreductase 1 (NQO1) signaling pathway, thus preventing dopaminergic neuron apoptosis and improving both exercise ability and anxiety behavior in mice (He and Bian, 2022). In MPTP-induced Parkinson’s model mice, catalpol exhibits anti-apoptotic, anti-oxidation, anti-inflammation, and promotes nerve regeneration. Its neuroprotective mechanism involves regulation of the Mitogen-activated protein kinase kinase (MK4)/JNK/C-Jun N-terminalkinase (C-Jun) signaling pathway (Wang et al., 2020; Wang et al., 2019).

Catalpol also exerts a neurorestorative effect after multiple cerebral infarctions (Huang Z. et al., 2024). In experiments with a middle cerebral artery occlusion model induced by nylon monofilament insertion and rat cortical neurons (RN-c MZ-7885) subjected to oxygen and glucose deprivation/reperfusion, catalpol activates the NRF1/K (lysine) acetyltransferase 2A (KAT2A)/methyltransferase like 3 (METTL3) axis and downregulates Beclin-1 expression, thereby relieving neuronal injury and excessive autophagy after cerebral ischemia (Liu K. et al., 2024). In a rat subarachnoid hemorrhage model created by intravascular perforation, catalpol promotes autophagy in nerve cells by activating the serine/threonine kinase (Raf)/mitogen activated protein kinase (MEK)/ERK signaling pathway, reducing apoptosis and neurological dysfunction, and improving brain injury in rats with subarachnoid hemorrhage (Meng Y. J. et al., 2023).

In a cerebral ischemia rat model established by electrocoagulation, catalpol promotes the proliferation of neural stem cells and immature neurons in the subventricular zone (SVZ) and protects the survival of mature neurons in the ischemic cortex and the dentate gyrus (DG) of the hippocampus via the vascular endothelial growth factor A (VEGF-A)/kinase insertion domain receptor (KDR) signaling pathway. Catalpol not only inhibits the over-proliferation of astrocytes in the ischemic cortex and DG, preventing neuroinflammation and scar formation from affecting the migration of newborn neurons, but also promotes recovery in the ischemic brain area by enhancing activation, providing neurotrophic substances, and supporting neuron survival, thus creating a favorable microenvironment for the migration of newborn neurons to the ischemic cortex area (Sun, 2022; Chen, 2021; Wang H. J., 2022; Wang H. J. et al., 2022; Sun et al., 2023). Catalpol promotes the proliferation and differentiation of neural stem cells into neurons after oxygen-glucose deprivation (OGD) through the VEGF-A/KDR-mediated Phosphatidylinositol 3-kinase (PI3K)/AKT/mTOR signaling pathway, thereby stimulating neurogenesis. As a multimodal neuroregenerative agent, catalpol also targets the insulin-like growth factor-1 (IGF-1) signaling pathway to drive axonal repair and functional recovery post-stroke (Liu et al., 2025). Delayed administration of catalpol enhances neural stem cell proliferation, reduces astrocyte proliferation, and restores neural function in the injured SVZ of rats with focal permanent cerebral ischemia induced by electrocoagulation. Even at a concentration as low as 10 μM, catalpol aids hippocampal neural stem cells in differentiating into neurons (Shao, 2020) and protects hippocampal neuron function (Xie et al., 2022). Catalpol also protects against hypoxic-ischemic brain damage by inhibiting ferroptosis through the PI3K/NRF2/system Xc-/GPX4 axis, reducing neuronal ferroptosis and ameliorating oxidative stress to protect the brain in neonatal rats (Lin et al., 2024). *In vivo* experiments using electrocoagulation and cultured neural stem cells from the hippocampus of newborn rats confirmed that catalpol activates the stromal cell-derived factor-1 (SDF-1)/C-X-C chemokine receptor 4 (CXCR4) and PI3K/AKT/ERK signaling pathways, promoting the migration of neural stem cells by microvascular endothelial cells after anoxia. Catalpol significantly enhanced the proliferation, migration, and differentiation of neural stem cells in the SVZ of cerebral ischemia rats, protected neurovascular units, and promoted the growth of neuronal axons (Zhang M. F., 2022; Wang H. J. et al., 2022; Zhang M. F. et al., 2024). Catalpol can also inhibit oxidative stress by promoting PI3K/AKT/mTOR signaling, alleviating neurological damage induced by thread embolism in rats with cerebral ischemia-reperfusion injury, and reducing brain edema (Qiao et al., 2024). In stroke rats induced by thread embolism, catalpol regulates VEGF and VEGFR to activate the Notch signaling pathway, promoting angiogenesis and neural function remodeling without increasing tissue edema (Sun L. P. et al., 2021). Furthermore, catalpol promotes exercise-mediated hippocampal neurogenesis by enhancing neural differentiation and the survival of mature neurons, thereby facilitating exercise-mediated brain functional changes in a post-traumatic stress disorder model (Sun L. et al., 2021).

**FIGURE 6:**
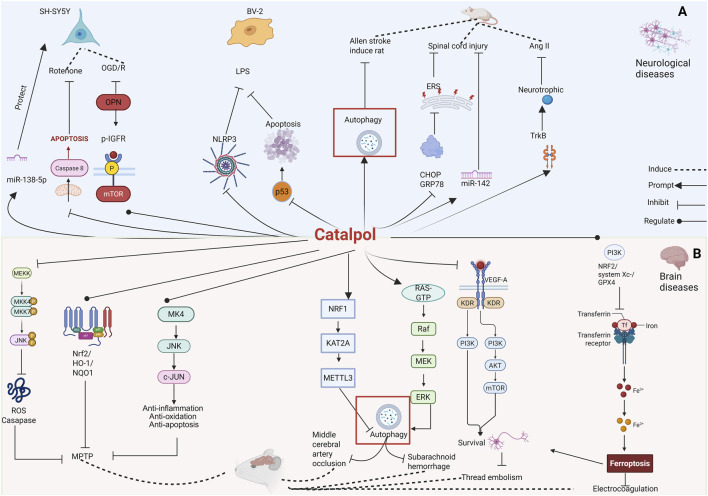
Effects of catalpol on the **(A)** neurological diseases and **(B)** brain diseases.

In summary, catalpol demonstrates significant potential as a disposal for brain diseases, including MPTP-induced Parkinson’s disease, middle cerebral artery occlusion, subarachnoid hemorrhage, cerebral ischemia induced by electrocoagulation, and neurological damage from thread embolism. Its promising results suggest it could be developed into a therapeutic drug for various brain diseases. Catalpol mainly exerts its effects through antioxidant activity, anti-apoptotic properties, regulation of autophagy, and promotion of neuronal survival, offering novel insights and a theoretical foundation for natural medicine in the settlement of brain diseases (Table 8; Figure 6).

**TABLE 8:** Pharmacological effects of catalpol in brain diseases.

Pharmacology	Experimental animal and dose	Experimental cell and dose	Effect	Ref.
Brain diseases	MPTP induced mice 15 mg/kg	—	↓ROS ↑SOD ↑Bcl2/BAX ↓cleaved Caspase-↓cleaved Caspase9 ↓p-MKK4/MKK4 ↓p-JNK/JNK ↓p-c-jun/c-jun	Wang et al. (2020)
	MPTP induced mice 15 mg/kg	—	↑HO1 ↑Nrf2 ↑Bcl2/BAX ↓ROS ↑SOD	Wang et al. (2019)
	MPTP induced mice 15 mg/kg	—	↑α-Synuclein ↑dopamine transporter ↓Bcl2/Bax ↑SOD1 ↓NLRX1 ↑GPX4 ↓GFAP ↓Iba1 ↓IL-1β ↓TNF-α ↓NLRP3 ↑Growth associated protein 43 ↑VEGF ↓p-MKK4 ↓p-JNK ↓p-c-Jun	Huang Z. et al. (2024); Liu W. et al. (2022)
	Nylon monofilament insertion to rats brain 5 mg/kg	Oxygen and glucose deprivation/reperfusion in rat cortical neurons cell RN-c, MZ-7885 10 μg/mL	↑NRF1 ↑met L3 ↓Beclin-1 ↓LC3II/I ↑p62	Sun (2022)
	Intravascular perforations in rat 10 mg/kg	—	↓Apoptosis rate ↓Bax ↑Bcl-2 ↑LC3-II ↑p-ERK ↑Beclin-1 ↑LC3-II ↑Raf ↑p-MEK/MEK ↑p-ERK1/2/ERK1/2	Chen (2021)
	Induction of rats by electrocoagulation 5 mg/kg	Isolation and culture of neural stem cells (NSCs) and brain microvascular endothelial cells (BMECs) from neonatal SD rats 1, 10, 100 μM	↓VEGF-A ↓p-KDR/KDR ↑Markers of neovascularization in ischemic cortex (CD105) ↑Neural stem cells in SVZ region Nestin ↑Immature neurons in SVZ region DCX ↑Mature neurons in ischemic cortex ↑Mature neurons in DG area of hippocampus ↓Astrocytes in ischemic cortex and DG area of hippocampus GFAP ↑PI3K ↑p-AKT/AKT ↑p-mTOR/mTOR	Wang R. R. et al. (2022); Wang J. et al. (2022); Sun et al. (2023); Liu et al. (2025); Shao (2020); Xie et al. (2022)
	Induction of rats by electrocoagulation 5, 10 mg/kg	Neural stem cells of neonatal rats 10 μM	↑Astrocyte number ↑Nestin ↑NeuN ↑DCX ↓GFAP	Lin et al., 2024; Zhang M. F. (2022)
	Induction of rats by electrocoagulation 5 mg/kg	Hypoxia-induced neural stem cells in hippocampus of rats 0.1, 1, 5, 10, 50 μM	↑Number of BrdU/DCX positive cells ↑MAP-2 ↑GAP-43 ↑SDF-1α ↑CXCR4 ↑PI3K ↑p-AKT ↑p-ERK ↑Migrating cell ↑Proliferation of neural stem cells	Zhang M. F. et al. (2024); Qiao et al. (2024)
	Induction of rats by Longa modified thread embolism method 9 mg/kg	—	↑VEGF ↑VEGFR ↑Notch1 ↑Notch4	Sun L. P. et al. (2021)

### 3.9 Neurological diseases

Prepared rehmannia root, as a representative Chinese herbal medicine of “supplying vital essence and marrow”, catalpol, its active component, has played multiple pharmacological roles in neurological diseases. Catalpol exhibits significant protective effects on human neuroblastoma SH-SY5Y cells induced by rotenone, with its mechanism linked to the enhancement of mitochondrial function and the reduction of apoptosis (Wan et al., 2020). Additionally, catalpol activates the Osteopontin/phosphorylation-insulin-like growth factor 1 receptor (p-IGFR)/mTOR signaling axis, promoting the vitality, migration, and axonal growth of SH-SY5Y cells following OGD/reoxygenation injury, contributing to neuronal protection, nerve cell migration, and axonal regeneration under ischemic and hypoxic conditions (Liu Y. et al., 2023).

Catalpol effectively alleviates cognitive impairment and neuropathological damage in isoflurane-exposed aged mice, showcasing its neuroprotective effects (Shi et al., 2022). In LPS-induced BV-2 microglia experiments, catalpol inhibits the activation of NLRP3 inflammasomes (Zhou, 2022; She et al., 2024) and blocks cortical neuronal oxidative damage by suppressing the p53-mediated Bcl-2/Bcl-2-associated X protein (Bax)/Caspase-3 apoptosis pathway and regulating the Kelch-like ECH-associated protein 1 (Keap1)/Nrf2 pathway, thus playing a pivotal role in reducing neuroinflammation (Yang C. et al., 2020).

In a rat model of traumatic brain injury induced by controlled cortical impact, catalpol provides neuroprotection against oxidative stress and neuroinflammation. It also ameliorates neurological impairment, blood-brain barrier disruption, cerebral edema, and neuronal apoptosis (Ni et al., 2023).

Catalpol inhibits apoptosis by enhancing autophagy, thereby reducing neuronal apoptosis and necrosis after acute spinal cord injury induced by Allen stroke in rats, promoting the recovery of motor function (Huang R. et al., 2023). In another spinal cord injury model induced by exposure of the T9 vertebral body, catalpol inhibits ERS by reducing the expression of CHOP and Glucose-regulated protein 78 (GRP78), ultimately protecting neurons and enhancing their survival (Huang Z. et al., 2022). In a weight-drop model of spinal cord injury, catalpol upregulates miR-142 and regulates the HMGB1/TLR4/NF-κB pathway, improving functional recovery (Xia et al., 2021).

Catalpol not only alleviates LPS-triggered cognitive impairment in mice post-sepsis (Hu W. et al., 2024), but also mitigates Ang II-induced blood-brain barrier damage. It shows high potential for treating hypertension-induced cerebral small vessel disease (Xia et al., 2022) by reversing neuroinflammation through blockade of the NF-κB pathway, upregulating neurotrophic factors via activation of the Tropomyosin Receptor Kinase B (TrkB) pathway, and preserving blood-brain barrier integrity.

Catalpol mitigates lead-induced neurotoxicity in PC12 cells by inhibiting the JAK2/STAT3 signaling pathway. Metabolomic analysis of PC12 cells, using ultra-performance liquid chromatography-quadrupole-time-of-flight mass spectrometry (UPLC-Q/TOF-MS), reveals that catalpol reverses an imbalanced metabolic state by regulating amino acid neurotransmitters, modulating amino acid and energy metabolism, and restoring oxidized substance levels, thus exerting neuroprotective effects (Zhang B. et al., 2024).

Catalpol also demonstrates neuroprotection in Alzheimer’s disease (Du et al., 2022; Tian et al., 2025). In a study using a lymphoblastoid cell line from patients with late-onset alzheimer’s disease, catalpol activates the Kelch-like ECH-associated protein 1 (Keap1)/Nrf2/antioxidant response element (ARE) signaling pathway, providing antioxidant and anti-apoptotic effects (Xiang et al., 2024). In both *in vitro* (human neuroblastoma SH-SY5Y cells) and *in vivo* (PSAPP-Tg: double transgenic mice expressing chimeric mouse/human amyloid precursor protein and mutant human presenilin 1 directed to CNS neurons), catalpol alleviates Alzheimer’s disease progression by promoting the level of miR-138-5p in exosomes secreted by neural stem cells (Meng S. et al., 2023). Catalpol enhances the expression of brain-derived neurotrophic factor (BDNF) by upregulating the phosphorylation of cAMP response element-binding protein (CREB), improving the survival of β-amyloid (Aβ)-damaged SH-SY5Y neuroblastoma cells, and providing a theoretical basis for its neuroprotective effects in Alzheimer’s disease (Huang W. Y. et al., 2023).

In summary, catalpol has demonstrated robust *in vitro* and *in vivo* neuroprotective activity against various neurological conditions, including those induced by rotenone, OGD/reoxygenation in SH-SY5Y cells, lead in PC12 cells, LPS in BV-2 cells, traumatic brain injury, spinal cord injury, cognitive impairment, and Alzheimer’s disease. These protective effects are primarily mediated through the reduction of apoptosis, combating oxidative stress and neuroinflammation, and alleviating ERS. This evidence supports catalpol as a promising candidate for the therapy of neurological disorders (Table 9; Figure 6).

**TABLE 9:** Pharmacological effects of catalpol in neurological diseases.

Pharmacology	Experimental animal and dose	Experimental cell and dose	Effect	Ref.
Neurological diseases	—	Rotenone induces human neuroblastoma cell SH-SY5Y 80, 400, 2000 nmol/L	↑MMP ↓α-synuclein ↓Bax/Bcl-2 ↓Caspase-3 ↓p-p38/p38	Shi et al. (2022)
	—	Oxygen-glucose deprivation/reoxygenation induce human neuroblastoma cell SH-SY5Y 50, 100, 200 μmol/L	↑GAP43 ↑OPN ↑p-IGFR ↑mTOR ↑phosphorylated ribosomal protein S6 ↓phosphatase and tensin homolog deleted on chromosome ten	Zhou (2022)
	—	LPS induces BV-2 microglia 10, 100, 1000 μM	↓NLRP3 ↓ASC ↓caspase-1 ↓IL-1β ↓IL-6 ↓IL-18	Yang L. et al. (2020); Ni et al. (2023); Huang X. X. et al. (2022)
	Induction of rats by Allen strike method 15, 30, 60 mg/kg	—	↓Bax ↑LC3-II ↑LC3 ↓p62	Xia et al. (2021)
	T9 segment vertebral body to expose the vertebral column induce rats 2 mg	Rat pheochromocytoma PC12 cells 5–80 uml	↓CHOP ↓GRP78 ↓Caspase-12 ↑Bcl-2 ↓Bax ↑NeuN ↑GAP43 ↑MAP-2	Hu W. et al. (2024); Xia et al. (2022)
	PSAPP-Tg mice 120 mg/kg	Human neuroblastoma SH-SY5Y cells 1, 10, 100 ng/mL	↑miR-138-5p ↑Caveolin-1	Meng S. et al. (2023)
	—	Aβ damages neuroblastoma cell SH-SY5Y 10 μM	↑BDNF ↑CREB	Huang W. Y et al. (2023)

### 3.10 Cancer

Catalpol often shows different mechanisms of action when intervening in different types of cancer. *In vitro*, catalpol has been shown to upregulate miR-34a in human colorectal cancer cell lines HCT116 and HT29, as well as in colorectal cancer samples and adjacent normal tissues from 60 patients with colorectal cancer. *In vivo*, using azoxymethane-induced colorectal cancer rats, catalpol reduced cell viability, suppressed autophagy, promoted apoptosis (Qiao et al., 2020). Catalpol also inhibits the migration and invasion abilities of colorectal cancer LOVO cells by blocking the activation of the FAK/MEK/ERK signaling pathway (Wang L. et al., 2025). In gastric cancer, catalpol inhibits the proliferation and migration of human gastric cancer cell HGC-27 induced by TGF-β1 by targeting the MAPK/ERK signaling pathway. This results in enhanced cell adhesion and suppression of the EMT process (Zhao, 2023). Catalpol-induced autophagy further promotes apoptosis in gastric cancer cells (Sun et al., 2021).

In MCF-7 breast cancer cells, catalpol inhibits cancer cell proliferation by inducing apoptosis via the mitochondrial apoptosis pathway and regulating protein post-translational modifications (Liu J. et al., 2022). *In vivo*, catalpol reduces tumor growth in nude mice, and its mechanism of action appears to involve the upregulation of FOXO3 and downregulation of FOXM1 expression (Zhang T. et al., 2023).

Catalpol also inhibits the proliferation and migration of oral squamous cell carcinoma cells and induces apoptosis by upregulating Keap1 and downregulating Nrf2 and HO-1 expression (Chen X. H. et al., 2024). In lung cancer, catalpol inhibits the survival of A-427 cells, hinders S-phase cell progression, and promotes apoptosis by increasing Bax and cleaved-caspase-3 levels. It also reduces the expression of programmed death-ligand 1 (PD-L1), enhances the survival of CD8^+^ T cells, and prevents immune escape of cancer cells (Zheng L. et al., 2024). In hepatocellular carcinoma (HCC), catalpol increases miR-140-5p expression in TGF-β1-induced HCCLM3 and Huh7 cells, inhibiting cell proliferation, migration, and EMT (Wu L. et al., 2021). Additionally, in HepG2 and HUH-7 HCC cell lines, catalpol significantly suppresses the PI3K/p-Akt/mTOR/NF-κB and VEGF/VEGFR2 signaling pathways, demonstrating potent anti-tumor effects against HCC (El-Hanboshy et al., 2021).

**FIGURE 7:**
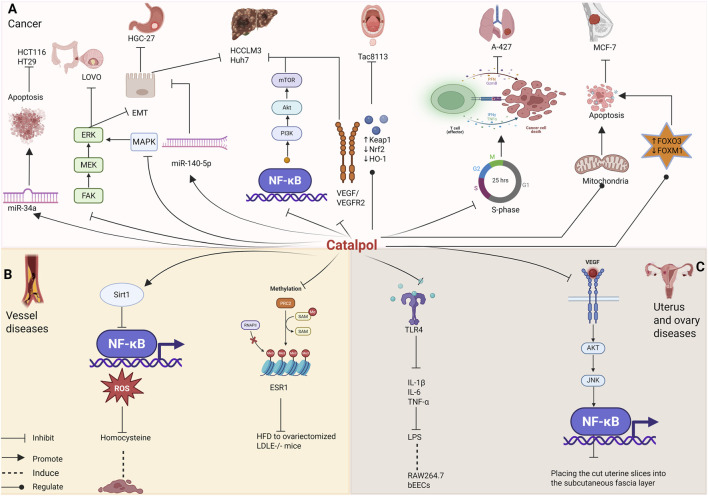
Effects of catalpol on the **(A)** cancer, **(B)** vessel diseases **(C)** uterus and ovary diseases.

In summary, catalpol exhibits clear anti-cancer effects across various human tumor types, including breast, liver, gastric, lung, colorectal cancers, and oral squamous cell carcinoma. It exerts anti-proliferative, pro-apoptotic, and EMT-inhibitory activities, making it a promising candidate with great medicinal value. These findings provide valuable insights for the development of cancer therapy and drug development (Table 10; Figure 7).

**TABLE 10:** Pharmacological effects of catalpol in cancer.

Pharmacology	Experimental animal and dose	Experimental cell and dose	Effect	Ref.
Cancer	Azoxymethane-induce rats 15 mg/kg	Azoxymethane-induce HCT116, HT29 20, 40 µM	↓Beclin 1 ↓LC3-II ↑miR-34a ↑Cytochrome c ↓Bcl-2	Sun et al. (2021)
	—	Colon cancer LOVO cell 60 μmol/L	↓Migration ability ↓Invasive ability ↓FAK ↓MEK ↓ERK ↓MRP1 ↓P-gp ↓PCNA ↓Vimentin ↓E-cadherin	Wang L. et al. (2025)
	—	TGF-β induces gastric cancer cell HGC-27 10, 25, 50, 100 μM	↑E-cadherin ↓Vimentin ↓N-cadherin ↓SNAI1 ↓ERK	Zhao (2023)
	Nude mice were injected with MCF-7 cells 20 mg/kg	Breast cancer MCF-7 cells 0, 5, 25, 50, 100, 200 μg/mL	↓Cell proliferation ability ↓S-phase cell ratio ↓G2/M phase cell ratio ↓ FOXO3 ↑Apoptosis rate ↑G0/G1 cell ratio ↑FOXM1 ↑caspase-3 ↑caspase-8	Zhang T. et al. (2023)
	—	Oral squamous cell carcinoma Tac8113 24 μg/mL	↑SOD ↓MDA ↓Nrf2 ↓HO⁃1 ↓Bcl⁃2 ↑Caspase3 ↑Keap1	Chen X. H. et al. (2024)
	—	Lung cancer cell A-427 1–20 μmol/L	↑Bax ↓Bcl-2 ↑Cleaved-caspase-3 ↑cGAS ↑STING ↑IFN-γ ↓IL-4 ↓IL-10 ↓TGF-β ↓PD-L1 ↓S-phase cell ratio ↑G0/G1 period proportion ↑G2/M phase cell ratio	Zheng L. et al. (2024)
	—	TGF-β induces HCCLM3 and Huh7 2.5, 5.0, 10.0, 20.0, 50.0, 100.0 μM	↓Viability ↓Proliferation ↓Invasion ↓Migration ↓Vimentin ↓N-cadherin ↑miR-140-5p	Wu, and Dum (2021)

### 3.11 Vessel diseases

Catalpol has a good preventive and therapeutic effect on atherosclerosis. Catalpol promotes angiogenesis in human umbilical vein endothelial cells (HUVECs) by upregulating angiogenesis-related proteins, suggesting its potential therapeutic application in vascular diseases (Ni J. et al., 2024). It can reduce the S-adenosylmethionine (SAM) levels, increase S-adenosine homocysteine (SAH), and decrease the SAM/SAH ratio by downregulating SAH expression. This modulation reduces the DNA methylation rate of Estrogen Receptor 1 (ESR1), upregulating the expression of estrogen receptor Erα. Catalpol also inhibits the abnormal increase of human vascular smooth muscle cells (HVSMC) induced by Angiotensin II, improving postmenopausal atherosclerosis in ovariectomized LDLE^−/−^ mice fed an HFD, effectively mitigating aortic disease in these mice (Zhang Y. H., 2022; Chen et al., 2021a; Chen et al., 2021b). Catalpol reduces apoptosis, oxidative stress, and inflammation by activating the expression of SIRT1 (He, 2022) and inhibiting the ROS/NF-κB signaling pathway (Wu et al., 2024b), thereby alleviating endothelial injury induced by homocysteine and playing a pivotal role in the development of atherosclerosis.

These findings suggest that catalpol has significant beneficial effects on atherosclerosis and other vascular-related diseases. The results highlight the potential of catalpol as an effective agent for treating vascular-related conditions (Table 11; Figure 7).

**TABLE 11:** Pharmacological effects of catalpol in vessel diseases.

Pharmacology	Experimental animal and dose	Experimental cell and dose	Effect	Ref.
Vessel diseases	Ovariectomized LDLE−/− mice with high-fat diet 7.22 mg/kg	AngII induces human vascular smooth muscle cells 20, 40,80 μm	↓TC ↓LDL-C ↓TG ↓DNMTs ↑ERs ↑SAH ↑G0/G1	Ni J. et al. (2024); Zhang H. W. et al. (2022); Chen et al. (2021a); Chen et al. (2021b)
	—	Homocysteine induces human aortic endothelial cells 5, 10 μm	↑Bcl-2/Bax ↑Sirt1 ↓ROS ↓P65	He (2022); Wu et al. (2024b)

### 3.12 Uterus and ovary diseases

At present, catalpol has been studied in the treatment of uterus and ovary diseases. In models of LPS-induced inflammation, including RAW264.7 cells, cow endometrial epithelial cells (bEECs), and a mouse endometritis model, catalpol inhibits the secretion and expression of inflammatory factors by targeting TLR4. This inhibition prevents the inflammatory process in RAW264.7 and bEECs and provides an anti-inflammatory effect in endometritis (Zhang H., 2021; Zhang H. et al., 2019). In a study involving uterine slices implanted into the subcutaneous fascia of rats and HeLa cells, catalpol inhibited angiogenesis in an endometriosis rat model by suppressing the activation of the Akt/JNK/NF-κB/VEGF pathway (Ma, 2024). Catalpol also mitigates the damage, oxidative stress, and apoptosis of ovarian tissue induced by Triptergium Glycosides in rats, regulating serum hormone levels and improving ovarian function (Ding et al., 2024).

In summary, catalpol offers significant benefits in treating uterine and ovarian-related diseases by inhibiting the secretion of inflammatory factors, blocking the Akt/JNK/NF-κB/VEGF pathway, and reducing oxidative stress and apoptosis (Table 12; Figure 7).

**TABLE 12:** Pharmacological effects of catalpol in uterus and ovary diseases.

Pharmacology	Experimental animal and dose	Experimental cell and dose	Effect	Ref.
Uerus and ovary diseases	Injection of LPS into mouse uterus horn 1, 10, 100 mg/kg	LPS induces RAW264. 7 cells 3, 0.3, 0.03 nM LPS induces endometrial epithelial cells (bEECs) in dairy cows 1, 0.1, 0.01 nM	↓IL-1β ↓IL-6 ↓TNF-α ↓TLR4 ↓IκBa ↓p-p65 ↓p38 ↓ERK ↓JNK ↓CXCL8 ↓CXCL5	Ma (2024)
	The cut uterine slices were placed in the subcutaneous fascia of rats 7, 14 mg/kg	HeLa cell 6.25, 12.5, 25, 50, 100, 200 μm	↓VEGF ↓p-Akt ↓p-JNK ↓p-P65	Zhang H. (2021); Zhang H. et al. (2019)
	Suspension of tripterygium glycosides induced rats 30, 60 mg/kg	—	↑Hormones estradiol ↓Luteinizing hormone ↓Follicle stimulating hormone ↓ROS ↓MDA ↑SOD ↑CAT ↑Bcl-2 ↑Shh ↑Gli1 ↓Cleaved Caspase-3 ↓Bax	Ding et al. (2024)

### 3.13 Other pharmacological effects

In addition to the previously discussed bioactivities, catalpol has been reported to treat a wide range of conditions, including hemorrhage, vitiligo, Sjogren’s syndrome, Guillain-Barré syndrome, chronic inflammatory pain, bone marrow suppression, thyroid dysfunction, aging, depression, pancreatitis, demyelination, burns, polycystic ovary syndrome (PCOS), Duchenne muscular dystrophy (DMD), allergic asthma, blood deficiency syndrome, and other diseases. Its therapeutic effects extend to various systems, including reproduction, the intestine, stem cells, skin, retinal pigment epithelium, and follicular development. Some of these effects are outlined below.

In a rat hemorrhage model established with 5% ethanol and dry yeast, catalpol intervention improved the pathological state of the rats (Ma, 2022). Catalpol also protects melanocytes from oxidative stress caused by ferroptosis induced by RSL-3, making it a potential settlement for vitiligo (Zhang, 2023).

In Sjogren’s syndrome, catalpol inhibits disease progression by regulating lnc-NONHSAT071210, which reduces the fine inflammatory response in salivary duct epithelium. This action decreases serum inflammatory factors and lymphocyte infiltration in the salivary glands of Sjogren’s syndrome model mice (He et al., 2024). Catalpol also enhances Schwann cell survival in response to damage induced by 2-methylpropionamidine dihydrochloride, offering a potential therapy strategy for Guillain-Barré syndrome (Li, 2020; Li et al., 2020). In a rat model of chronic inflammatory pain induced by complete Freund’s adjuvant, catalpol reduced pain behaviors by modulating the HDAC4/PPAR-γ signaling pathway. It decreased the expression of the NF-κB/NLRP3 inflammatory axis in the spinal cord, effectively reducing mechanical allodynia and thermal hyperalgesia when administered intrathecally (Zhao B. et al., 2022).

Catalpol has been shown to counteract bone marrow suppression induced by acetylphenylhydrazine and cyclophosphamide in rats (Liu K. X. et al., 2022). It outperforms methimazole in treating hyperthyroidism in mice and reduces oxidative stress damage to the liver (Yang L. M. et al., 2021). In aging models using the mutant insulin-like receptor gene (daf-2, CF1041) and mammalian transcription factor family protein homologs (daf-16, CF1038), catalpol enhanced antioxidant gene expression and the antioxidant response, thus delaying aging by activating the insulin/IGF-1 signaling pathway in nematodes (Zhao et al., 2024; Zhao et al., 2023).

Based on the spectrum-effect relationship and activity verification, catalpol has been shown to exert antidepressant effects (Hu C. et al., 2024). In chronic unpredictable mild stress mice, catalpol alleviates depressive symptoms by downregulating the oxidative stress-mediated activation of the NLRP3 inflammasome and neuroinflammation (Wang Y. L. et al., 2021; Liang et al., 2023). Additionally, catalpol mitigates depressive-like behavior in mice with pathological hyperglycemia, with its antidepressant mechanism partly attributed to the upregulation of the PI3K/AKT/Nrf2/HO-1 signaling pathway. This restores the balance between oxidative and antioxidant damage, contributing to its therapeutic effects (Wu X. et al., 2021; Wang J. et al., 2021). In L-thyroxine-induced depression in mice, catalpol inhibited the Cyclooxygenase-2/NLRP3 signaling pathway, reducing neuronal damage and exhibiting antidepressant effects. Additionally, catalpol showed high sensitivity in improving hippocampal SIRT1-mediated synaptic plasticity and neurogenesis in females. It enhanced synaptic plasticity and alleviated depressive behaviors, with the action pathway involving Tyrosine kinase receptor B signaling (Song et al., 2021; Wu et al., 2023; Wu X. et al., 2022; Wu et al., 2024c; Wu et al., 2024d). Catalpol also improves depression-like behavior induced by middle cerebral artery occlusion combined with chronic unpredictable mild stress in rats (Wang X. Y. et al., 2022), potentially by regulating the central serotonin system and promoting BDNF secretion (Wu X. et al., 2022).

In a severe acute pancreatitis rat model induced by sodium taurocholate solution, catalpol demonstrates therapeutic effects on intestinal inflammation by downregulating the HMGB1/RAGE signaling pathway (Huang W. L. et al., 2024).

As a promising candidate for demyelinating diseases, catalpol promotes oligodendrocyte generation and myelin repair, alleviates clinical neurological dysfunction, inhibits inflammatory infiltration, and increases the proportion of Treg cells (Wu M. et al., 2022; Yuan et al., 2015). Catalpol also reduces apoptosis and proliferation following vascular injury in an HUVE cell line subjected to OGD, suggesting its potential as a burn therapy agent (Ni J. R. et al., 2024). In PCOS, catalpol upregulates SIRT1 levels and inhibits the activation of the NF-κB signaling pathway in dehydroepiandrosterone-induced rats and human ovarian granulosa cell line KGN (Zhao Z,J. et al., 2022), demonstrating its therapeutic potential for PCOS. In DMD, a progressive neuromuscular disorder caused by mutations in the dystrophin gene, catalpol attenuates muscle fibrosis by inhibiting the TGF-β1/TAK1 signaling pathway. It restores skeletal muscle strength and alleviates skeletal muscle damage in aged dystrophin-deficient mice (Xu et al., 2021; Xu et al., 2020). Catalpol also has suppressive effects on IgE/ovalbumin (OVA)-induced asthma in mice. It inhibits the degranulation of bone marrow-derived mast cells and reduces the recruitment of mast cells while increasing mucus production in lung tissues, thus benefiting the treatment of asthma (Chiu et al., 2021). Using 16S rRNA gene sequencing combined with serum and spleen metabolomics, catalpol was shown to exert its therapeutic effects on blood deficiency syndrome via glycerophospholipid and sphingolipid metabolism pathways (Zhang W. et al., 2023). In LPS/carbonyl cyanide 3-chlorophenylhydrazone (CCCP)-induced fever models in rats, catalpol undergoes deglycation *in vivo*, where its hemiacetal group covalently binds to Lys239 of UCP2 in the mitochondria of the liver via an ɛ-amine nucleophilic addition. This interaction affects proton leakage, improves mitochondrial membrane potential, and enhances ADP/ATP transformation efficiency, leading to an antipyretic effect (Shen et al., 2024).

Catalpol enhances mitochondrial function and antioxidant capacity in germ cells, improving the efficiency of *in vitro* maturation and their subsequent embryonic development potential (Xu, 2023; Wang et al., 2023). It protects granulosa cells from H_2_O_2_-induced oxidative damage and apoptosis by activating the PI3K/Akt/mTOR signaling pathway, providing a potential therapeutic approach for regulating disrupted follicular development (Yan et al., 2020).

Catalpol also increases the levels of gut bacterial metabolites, particularly short-chain fatty acids (Jing et al., 2025). In an LPS-induced inflammatory response model using intestinal epithelial cell-6, catalpol mitigates inflammation by activating the AMPK/mTOR signaling pathway, reducing the release of inflammatory factors, alleviating cellular oxidative stress, and inhibiting cell apoptosis (Gao et al., 2023).

By optimizing the ischemic-hypoxic microenvironment and regulating paracrine actions, catalpol promotes the proliferation and differentiation of MSCs (Zhang J. S. et al., 2021). It protects dermal fibroblasts from oxidation and apoptosis, thereby promoting skin wound healing (Lang et al., 2024). Catalpol also improves atopic dermatitis (Sun et al., 2024). In male “McFarlane flap” rat experiments, catalpol enhances the viability of random skin flaps by activating the SIRT1-mediated autophagy pathway (Jiang et al., 2022; Ma et al., 2024). Additionally, catalpol protects retinal pigment epithelial ARPE-19 cells from oxidative stress through activation of the Keap1/Nrf2/ARE pathway and the inactivation of oxidative stress-mediated apoptotic pathways (You et al., 2021).

## 4 Safety

The maximum dose of catalpol (50 mg/mL) was administered to mice via gavage over 2 weeks, with no signs of acute toxicity or mortality, suggesting that catalpol is safe and non-toxic (Dong et al., 2009). Biodegradable mesoporous silica nanoparticles loaded with catalpol were injected into the joint cavity of rats. Histopathological analysis of tissues from the liver, spleen, kidney, lung, and heart revealed no abnormalities, and biochemical assessments showed no signs of hepatorenal toxicity (Zhou et al., 2025). In 2017, the new hypoglycemic drug “Catalpol Tablets” received approval for clinical trials by the China State Food and Drug Administration. Recent research from the Science and Technology Department of Qinghai Province, China, found that catalpol is rapidly absorbed in patients, with an absolute bioavailability of 66.9%. The compound demonstrates a quick onset, rapid and complete excretion, no accumulation in the body, and no damage to major organs. These properties make it suitable for long-term use without significant drug interactions, positioning it as a TCM hypoglycemic agent with minimal toxicity and side effects.

The definite toxicity, safety margin and potential adverse reactions have not been reported. Regarding safety, compelling insights emerged from our retrieval. The similarity between zebrafish and human genes is as high as 70%–87%, and about 84% of known human disease-related genes can find homologous genes in zebrafish genome. This means that many genes related to human diseases have similar functions and mechanisms in zebrafish. The experiment confirmed that catalpol did not show genotoxicity and teratogenicity at the dose of 25 μ mol/L (Wang D. D. et al., 2025; Wu et al, 2019). In a study on the efficacy and safety of catalpol in the treatment of postoperative patients with locally advanced colon cancer, patients treated with an intraperitoneal injection of 10 mg/kg catalpol twice a day for 12 weeks, monitoring patients appeared with adverse reactions of diarrhea, nausea, vomiting, gastrointestinal ulcers, allergy, constipation, alopecia, and peripheral neurotoxicity. Only non-fatal adverse effects occurred in the catalpol treatment group, showed benefits in clinical outcome, and with no serious complications (Fei et al., 2018). Attention should be paid to safety data regarding the use of catalpol during pregnancy or breastfeeding, teratogenicity and other issues. In the future, it is hoped that researchers should pay attention to and disclose relevant data in the future, so as to better promote the development of catalpol.

## 5 New dosage forms

Catalpol, a small-molecule drug, boasts high water solubility and is easily administered orally; however, it faces limitations such as low fat solubility, difficulty crossing the blood-brain barrier, and a short half-life. These characteristics necessitate structural modifications to enhance its pharmacological activity (Table 13) (Pungitore et al., 2007).

**TABLE 13:** New dosage form of catalpol.

Experimental model: cell	Experimental model: animal	Experimental model: clinical	Preparation method	Ref.
STZ induced SH-SY5Y cells	—	—	Fully dissolving catalpol with pyridine, adding propionic anhydride, placing in a microwave reactor, reasonably adjusting the stirring rate, diluting the reaction solution with CH2Cl2 after the reaction, vacuum concentrating on a rotary evaporator to remove pyridine, then adding ethyl acetate for extraction, drying, filtering and concentrating the organic phase to obtain the catalpol propionylation product	Zhang Q. X. et al. (2021); Liu et al. (2020)
MC3T3-E1 cells	Establishment of rat model of femoral condyle defect of right knee joint	—	The activated titanium tablets were put into dopamine tris-hcl buffer, soaked in dark for 24 h, washed with distilled water, dried in a 20 °C oven for 30 min, put into catalpol tris-hcl buffer and soaked for 12 h. After completion, rinse with distilled water, and then dry in a 20 °C oven for 30 min	Li (2021)
RAW264.7 BMSCs	Construction of rat model of subcutaneous heterotopic implantation of fibrous membrane	—	Polylactic acid and gelatin were added into trifluoroethanol, and the same spinning solution was prepared for spinning. EDC and NHS were dissolved in the mixed solvent of ethanol and deionized water to submerge the fiber membrane for 12 h, and then freeze-dried by a cold dryer	Zhang Y. L. (2021); Zhang Y. et al. (2022)
Murine preosteoblast cell line MC3T3-E1	Titanium implants were surgically implanted into the femoral condyle defects in normal mice and mouse models of type 2 diabetes mellitus	—	Sodium hyaluronate solution was prepared in ddH2O, and the Titanium-poly (ethylenimine) substrate was immersed in the hyaluronate solution to obtain the titanium-poly (ethylenimine)-hyaluronate substrate. Subsequently, chitosan was dissolved in water containing 1% v/v glacial acetic acid. Then, chitosan-catalpol solutions with concentration. The Titanium-poly (ethylenimine) substrate was im-poly (ethylenimine)-hyaluronate substrates were then immersed in chitosan solutions with the indicated concentrations of catalpol air-dried	Zhang Y. et al. (2024); Yang et al. (2024)
Esophageal cancer cells Eca-109 and EC-9706 Pancreatic cancer cells PANC-1, BxPC-3 Normal pancreatic cell line HPDE6-C7	—	—	The ratio of n (catalpol):n (iodine):n (triphenylphosphine):n (imidazole) was 1:6:6:12, the solvent was ultra-dry tetrahydrofuran, the reaction temperature was 0 °C. A series of C10-position pyrazole modified catalpol derivatives (3a–3 m) were synthesized by C10-iodocatalpol treated with different substituted pyrazole derivatives under K2CO3 in DMF at 70 °C	Kong et al. (2023a); Kong et al. (2023b)
—	—	25 female volunteers, under 30 years of age (selection criteria: Normal skin without any dermatological skin lesions)	Lipid nanoparticles were produced using a modified emulsification–ultrasonication method based on multiple emulsion, introducing the dispersions of lipid nanoparticles (without active ingredients or with catalpol) into hydrogel formulations. Then, the lipid nanoparticle dispersion (in a ratio of 50:50 wt%) was added, upon intensive stirring until the desired consistency was obtained. The resulting cosmetic formulation was stirred for another 10 min to stabilize the consistency	Dąbrowska and Nowak (2021)

By using catalpol and propionyl anhydride as raw materials, with pyridine as the solvent and acid-binding agent, catalpol perpropionyl was synthesized. This modified compound effectively reverses the decrease in SH-SY5Y cell activity induced by STZ, demonstrating neuroprotective effects and presenting itself as a potential neuroprotective agent (Zhang Q. X. et al., 2021; Liu et al., 2020). Additionally, dopamine-loaded catalpol on titanium implants, when co-cultured with osteoblast MC3T3-E1 cells, promoted cell proliferation, adhesion, and enhanced osteoblast proliferation, differentiation, and mineralization. In a rat femoral condyle defect model, catalpol-loaded titanium implants significantly promoted bone formation and improved bone integration with the implant (Li, 2021). Catalpol, loaded onto hydrophilic electrospun polylactic acid/gelatin fiber membranes (PLLA/Gel), was implanted under the skin of rats for 2 months. The introduction of catalpol alleviated the inflammatory reaction caused by subcutaneous implantation of the fiber membrane, inducing a shift from M1 to M2 phenotype macrophages. This led to improved ectopic bone formation and inhibited osteoclast formation (Zhang Y. L., 2021; Zhang Y. et al., 2022). Furthermore, using a layer-by-layer electroassembly technique, catalpol-containing hyaluronic acid/chitosan multilayers were deposited onto titanium implants. In murine preosteoblast MC3T3-E1 cells and in a rat femoral intramedullary implantation model, catalpol-coated titanium implants enhanced early-stage osseointegration, reducing the failure rate of internal fixation in osteoporotic fractures (Zhang Y. et al., 2024; Yang et al., 2024).

Regarding anticancer activities, catalpol underwent pyrazole heterocyclic modification at the C10-position hydroxyl group, yielding derivatives that demonstrated strong inhibitory activity against esophageal cancer cells (Kong et al., 2023a). C10-position imidazole-modified catalpol derivatives also showed significant inhibitory effects on the growth of human pancreatic cancer cells (PANC-1 and BxPC-3), with a 91.6% efficacy against BxPC-3 and 73.1% efficacy against PANC-1 (Kong et al., 2023b). Additionally, catalpol hexapropionate (CP-6) has been designed and synthesized as an anti-aging drug (Cheng X. et al., 2020).

Lipid nanocarriers were created using an emulsification-ultrasonication method based on multiple emulsions. In experiments involving 25 female volunteers, catalpol applied in the form of lipid nanoparticles demonstrated anti-wrinkle, moisturizing effects, and regeneration of the protective barrier of the stratum corneum (Dąbrowska and Nowak, 2021).

## 6 Novel drug delivery routes

However, catalpol is easily degraded in the normal gastrointestinal environment, requiring oral doses several times higher than intravenous administration. Therefore, improving its absorption for more effective application is crucial. In a mouse model of cerebral ischemia induced by thread embolism, nasal administration of catalpol maximized its anti-hypoxia injury effects, effectively protecting against brain damage in rats with acute cerebral ischemia and improving neurological function (Wang J. H., 2022; Wang J. et al., 2022).

## 7 Conclusion

This review provides a comprehensive overview of the pharmacological activity, safety, novel dosage forms, and innovative drug delivery routes of catalpol. As an extract from the natural plant *Rehmannia glutinosa* Libosch, catalpol exhibits protective effects on various organs and tissues, both *in vivo* and *in vitro*. Its mechanisms of action are primarily related to anti-inflammatory, anti-oxidative stress, pro-apoptotic and anti-apoptotic effects, anti-fibrosis, autophagy regulation, anti-ERS, and metabolic regulation. Notably, catalpol influences various signaling pathways, cells, and cytokines, enabling it to exert its therapeutic effects across multiple systems. Catalpol demonstrates a broad pharmacological spectrum, addressing a wide range of diseases, including those affecting the kidneys, diabetes, arthritis, bones, brain, nervous system, heart, liver, and lungs. It also shows significant efficacy against other conditions such as cancer, vascular diseases, and reproductive health issues.

Catalpol, as the main active ingredient of *Rehmannia glutinosa* Libosch, has entered the clinical trial stage and is expected to become an innovative drug for the treatment of diabetes and related complications. Its clinical research has made rapid progress, and its effectiveness have been supported by more clinical data, which has greater clinical transformation potential. At present, metformin is the main treatment for diabetes, but the accumulation of metformin may cause metabolic and nutritional disorders, nervous system abnormalities, gastrointestinal abnormalities, liver and gallbladder dysfunction and skin and subcutaneous tissue abnormalities. In terms of natural products, similar iridoid components, such as β-dihydroplumericin A isolated from frangipani and Neocornusides A isolated from calendula, all have the effect of treating diabetes. But compared with catalpol, these compounds are still in the basic pharmacological research stage, and the transformation path from basic research to clinical trials needs a long time to explore and improve (Fei et al., 2024).

**FIGURE 8:**
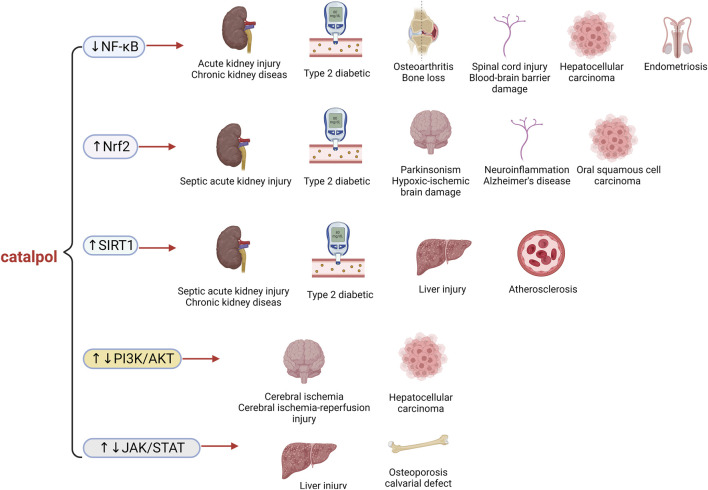
The main mechanisms of catalpol are mapped to different disease environments.

The main mechanisms of catalpol are mapped to different disease environments, showing similar regulatory effects, as shown in the Figure 8. Among them, NF-κB, Nrf2, SIRT1, PI3K/AKT/mTOR and JAK/STAT are the key ways for future pharmacological research of catalpol. Catalpol can treat kidney diseases, diabetes and nervous system diseases by inhibiting NF-κB pathway and up-regulating Nrf2 and SIRT1 pathway. Catalpol plays a significant role in the treatment of brain and cancer through PI3K/AKT/mTOR pathway. In addition, catalpol inhibits JAK/STAT signaling pathway to reduce neurotoxicity and liver injury, and promotes osteogenic bone regeneration and angiogenesis by activating JAK2/STAT3 axis in BMSC and rats. In short, the complexity of these pathways may lead to inconsistent therapeutic effects and unexpected interactions. While catalpol, as an extract from TCM herbs, holds significant therapeutic potential, further research is needed to fully harness its capabilities in clinical applications. Research and development team of catalpol tablets utilizing liquid scintillation counting, UHPLC-β-ram, and UHPLC-Q-Exactive Plus MS have shown that catalpol is primarily excreted through urine, with drug-related substances concentrated in the stomach, large intestine, bladder, and kidneys. The metabolism of catalpol in rats is mainly mediated by the intestinal microbiota, resulting in the formation of an aglycone-containing hemiacetal hydroxyl structure (Liu, 2014). The research and development team then conducted a phase IIa clinical trial of catalpol tablets, completed the pharmacokinetic study of catalpol tablets in patients with type 2 diabetes mellitus, and found that the drug half-life of catalpol was 1.5–2.5 h, Tmax was 1.5–2 h, and Cmax was 3 h. When catalpol was administered at 100 mg and 400 mg, the excretion rate in urine was 1.17 ± 1.00 and 1.04 ± 0.89 respectively, and the urine excretion was 1.17 ± 1.00 and 4.17 ± 3.55 respectively. When the dosage was 800 mg, the fecal excretion rate was 17.79 ± 35.29 and the fecal excretion was 142.30 ± 282.32. The efficacy and safety of catalpol tablets in patients with type 2 diabetes were studied, and the dosage was determined to be 900 mg catalpol tablets +300 mg placebo in the low-dose group and 1200 mg catalpol tablets in the high-dose group (Xining science and technology bureau., 2022). Further investigations revealed that catalpol is unstable in the gastrointestinal tract, but its bioavailability, when administered via gavage, reaches nearly 50%. This bioavailability is approximately ten times greater compared to intravenous administration, especially in the context of diabetes treatment. This difference may be due to metabolites produced by catalpol *in vivo* (Ge et al., 2023).

At present, the research field of catalpol has developed from the initial extraction and determination of active ingredients to the mechanism research of basic experiments, and then to the clinical research and new drug development in recent years, showing the transformation process from traditional Chinese medicine monomer to clinical application. Some progress has been made in many aspects, from the cellular level to the molecular biological level, from experiment to clinic, catalpol shows considerable therapeutic promise, but its clinical application is limited by the intricate biochemical pathways it affects. As far as the current research progress is concerned, catalpol still has some shortcomings in metabolic pathway and bioavailability *in vivo*. Moreover, the extraction route of catalpol is limited, which is not conducive to separation because of its unstable chemical properties. In the subsequent development, it is necessary to further study the dosage form and optimize the existing processing technology to make it into a clinical dosage form. At present, most of the research is based on animal models and *in vitro* experiments, and a few pharmacological effects have been clinically confirmed, especially the hypoglycemic effect of catalpol has been verified in the endocrine field, catalpol tablets have entered the phase 2 clinical study (CTR20220555) in China, aiming at evaluating the pharmacokinetics, efficacy and safety of catalpol tablets in patients with type 2 diabetes, small molecular monomer drugs have begun to face the clinic with suitable dosage forms. However, the limitations of some research results, including insufficient data and small sample size, may lead to limited reliability and universality of the results. Future research should focus on further elucidating its detailed mechanisms of action and developing targeted pathways, potentially enhancing catalpol’s effectiveness in clinical settings. Different drug absorption models can be used to explore the absorption and transport characteristics of catalpol, so as to understand its absorption and transport mechanism more deeply and comprehensively, and provide a solid theoretical basis for the clinical design of catalpol drug dosage forms. we should focus on solving the challenges of bioavailability, dosing, and translation into clinical use.

Natural active substances are a significant source of new drug development. With the ongoing research into catalpol, clinical studies focused on chemical structure modifications and derivative compounds have emerged. In 2007, fully acetylated derivatives of catalpol were shown to inhibit ileal contraction induced by acetylcholine in guinea pigs (Fleer and Verspohl, 2007). In 2015, a catalpol derivative—6-O-catalpol—was found to have anti-inflammatory effects, inhibiting protein kinase C activity, which may be linked to the inactivation of extracellular signal-regulated kinase and NF-κB in the downstream inflammatory signaling pathway of human monocytes/macrophages (Le et al., 2015). Currently, new dosage forms and administration routes for catalpol include titanium-implanted catalpol, PLLA/gel-loaded catalpol, pyrazole heterocyclic modification at the C10-position hydroxyl of catalpol, catalpol hexapropionate (CP-6), catalpol lipid nanocarriers, catalpol freeze-dried powder injection (He, 2009), catalpol nasal drops, and catalpol gel (Zhu, 2019). A croton acylated catalpol derivative (patent batch number CN 108912183 A) demonstrates promising anti-aging activity, enhances blood-brain barrier permeability, and achieves an esterification yield of 99.16%. These findings suggest that future developments of catalpol should not only include intramuscular and intravenous injections but also consider novel oral dosage forms to increase its absorption rate and bioavailability.

With the advent of new methods and technologies, the medicinal value of catalpol has significantly increased. Catalpol tablets have become the first Class 1 new Chinese medicine for diabetes in China. In 2022, catalpol was classified as Class 1.2 clinical by China Suzhou Yusen New Drug Development Co., Ltd., and is now recognized for its potential to improve cerebral ischemia injury. In the same year, with support from the Science and Technology Bureau of Xining City, China, and undertaken by Qinghai Yangzong Pharmaceutical Co., Ltd., the Phase IIa clinical study of Catalpol, a new Chinese medicine, passed acceptance and evaluation. This study on the pharmacokinetics of catalpol tablets in patients with type 2 diabetes, along with the investigation into its effectiveness and safety at different doses, contributed to the determination of its clinical, pharmacological, and pharmacodynamic properties. These results, which detail the distribution, metabolism, and dosage of catalpol, have reached an advanced level in China.

The latest research of Academician Luqi Huang’s team has identified the key cyclooxygenase gene involved in catalpol synthesis for the first time through telomere-to-telomere (T2T) genome assembly. This breakthrough has laid the foundation for analyzing the biosynthetic pathway of iridoid glycosides and provided genomic support for quality control, synthetic biological development (such as catalpol production via microbial fermentation), and the revision of species taxonomy in *Rehmannia glutinosa* (Wang F. et al., 2025).

Given its multiple pharmacological activities and safety profile, catalpol holds significant advantages in the settlement of various diseases. It is reasonable to believe that through further in-depth animal and cell studies, along with standardized clinical research, catalpol will continue to show promise in organ and tissue protection. Unlocking its full therapeutic potential will provide convincing evidence for more effective and safer interventions in healthcare.
